# Immunoglobulin heavy chain locus duplication in bats

**DOI:** 10.1126/sciadv.aeb6714

**Published:** 2026-07-29

**Authors:** Taylor Pursell, Ashley Reers, Artem Mikelov, Prasanti Kotagiri, Brandon Lam, James A. Ellison, Scott D. Boyd, Hannah K. Frank

**Affiliations:** ^1^ Department of Pathology, Stanford University, Stanford, CA 94305, USA.; ^2^ Department of Microbiology & Immunology, Stanford University, Stanford, CA 94305, USA.; ^3^ School of Science & Engineering, Tulane University, New Orleans, LA 70118, USA.; ^4^ Department of Immunology and Pathology, Monash University, Melbourne, Australia.; ^5^ Poxvirus and Rabies Branch, Division of High-Consequence Pathogens and Pathology, Centers for Disease Control and Prevention, Atlanta, GA 30329, USA.; ^6^ Sean N. Parker Center for Allergy and Asthma Research, Stanford University, Stanford, CA 94305, USA.

## Abstract

Bats are major reservoirs of viruses that can be transmitted to humans in zoonotic outbreaks. Antibody-mediated immunity plays an important role in shaping viral evolution and immune evasion but remains understudied in bats. All known mammals have a single immunoglobulin heavy chain (IgH) gene locus and up to two light chain loci. We have identified dual IgH loci on separate chromosomes in 26 bat species, highlighting extreme variation of immunogenetic architecture in order Chiroptera. In a model species, *Eptesicus fuscus*, we leveraged single-cell transcriptomes to confirm functional rearrangement and expression of both loci, but with different mechanisms for generating antibody diversity and function. These results provide a foundation for analysis of humoral immunity and pathogen response in bats.

## Teaser

Some bats, unlike other mammals, have two functional antibody heavy chain loci that may affect how they respond to pathogens.

## INTRODUCTION

Antibodies play a large role in shaping viral evolution (*1*). By exerting selective pressure, they drive mutations in viral surface proteins, leading to changes in entry receptors (*2*) and the emergence of immune escape variants (*3*). Bats, unique in their ability to host a vast array of current and emerging zoonotic viruses (*4*), mount humoral responses to a range of viruses as evidenced by serological studies (*5*–*9*). Despite this, the genetic and molecular basis for the formation and evolution of bat B cell receptor (BCR) repertoires and antibody responses is largely uncharacterized. Understanding bat humoral immunity is an underexplored way of approaching spillover risk (*10*), given bats’ role as reservoir hosts.

Current mammalian immunological data show that the genes that generate the heavy and light chains of immunoglobulin (Ig) protein complexes are encoded by a single heavy chain locus and up to two light chain loci, designated kappa and lambda. Each of these loci contains arrays of variable (IGHV/IGKV/IGLV), diversity (IGHD), joining (IGHJ/IGKJ/IGLJ), and constant (IGHC/IGKC/IGLC) genes. The number, diversity, and organization of these genes contribute to the resulting Ig repertoire, which varies across species. There are over 1500 known species of bats with diverse biology and modes of living. Despite this species diversity, studies characterizing Ig genes in bats have been limited to a few species [e.g., *Pteropus* spp. (*11*, *12*), *Rousettus aegyptiacus* (*13*), and *Rhinolophus* spp. (*14*)]. To address this gap, we conducted comprehensive germline genetic annotation and evolutionary analysis of Ig loci in a subset of bats from the largest family, Vespertilionidae.

## RESULTS

**Fig. 1.**
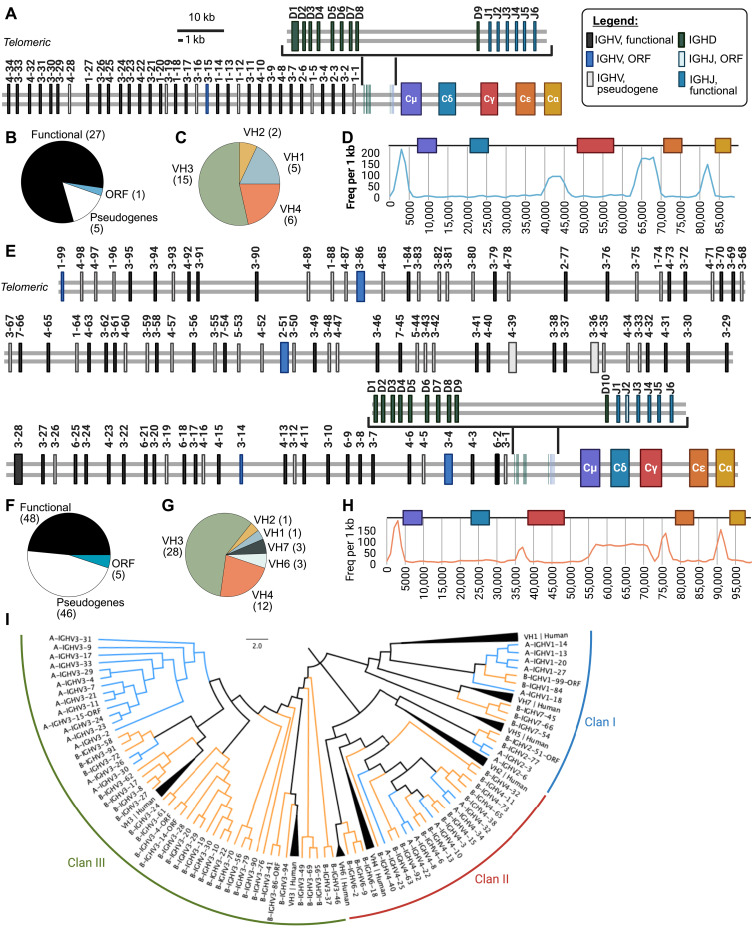
Genomic annotations and analysis for both IGH loci. (**A**) Schematic of Ig gene annotations for A-IGH locus. (**B**) Pie chart of functionality and (**C**) gene family distribution for functional variable (V) genes for A-IGH locus. (**D**) Density plot of AID hotspots per kilobase in the constant gene encoding regions for A-IGH locus. (**E**) Schematic of Ig gene annotations for B-IGH locus. (**F**) Pie chart of functionality and (**G**) gene family distribution for functional variable genes for B-IGH locus. (**H**) Density plot of AID hotspots per kilobase in the constant gene encoding regions for B-IGH locus. (**I**) Nearest neighbor-joining tree of functional and ORF V genes for A-IGH and B-IGH with human V genes collapsed by V-gene family for reference. Created in BioRender. Pursell, T. (2026) https://BioRender.com/rru8vm2.

We identified two Ig heavy chain (IgH) loci in a high-quality, long-read genome assembly of *Eptesicus fuscus* [National Center for Biotechnology Information (NCBI) genome assembly DD_ASM_mEF_20220401] (Fig. 1). No other mammal was previously known to have two IgH loci, although IgH duplication has been described in some fish (*15*). The smaller locus (IGH locus A, A-IGH) spans 272 kb on chromosome 5, and the other (IGH locus B, B-IGH) spans 918 kb on chromosome 24. The overall structure and orientation of both loci mirror that of humans with sequential arrays of variable (V_H_), diversity (D), and joining (J_H_) gene segments followed by a single, functional copy of each of the five major mammalian constant region genes (C_H_) (Fig. 1, A and E, and tables S1 and S2). No flanking genes appear to have been transposed as A-IGH is flanked by *TMEM212* and *CRIP1*, both genes known to flank the IGH locus in other mammals, while B-IGH is flanked by *MXL*.

Examination of the constant region genes in both loci identified IGHM, IGHD, IGHG, IGHE, and IGHA with conserved ordering and exon structure. Recombination and isotype switching in mammals is initiated by cytidine deamination mediated by the activation-induced cytidine deaminase enzyme (AID), which preferentially uses the 5′-AGCT-3′ motif (*16*–*18*). Increased density of such switch motifs immediately upstream of the CH1 domains for each IGHM, IGHG, IGHE, and IGHA in both loci (Fig. 1, D and H) was observed, suggesting conserved mechanisms for AID-dependent class switching. Similar to humans, there is no switch region between genes coding for IgM and IgD, consistent with alternative splicing for coexpression of these isotypes (*19*–*21*). B-IGH has a 17.5-kb region with increased switch motif count between B-IGHG and B-IGHE (Fig. 1H) that is not observed in A-IGH (Fig. 1D), which could affect the rate and efficiency of class switching at this locus.

The IGHJ gene cluster is composed of six functional genes on A-IGH and five functional genes and one open reading frame (ORF) on B-IGH (table S3), with all segments encoding the canonical di-glycine bulge WGXG motif and the upstream recombination signal sequence (RSS) containing a 23–base pair spacer. Except the ORF, all interchromosome homologs share at least 90% nucleotide identity (fig. S1A).

We identified 10 IGHDs on A-IGH and 9 on B-IGH (table S4) using homology and RSS searching in the intergenomic space between the most 3′ IGHV and the most 5′ IGHJ. The IGHDs are more divergent than IGHJs between the loci, with only two gene pairs sharing >90% homology (fig. S1B). Thus, with the duplication, the effective germline IGHD diversity is only slightly less than that of the human locus, which has 27 germline IGHDs.

While A-IGH encodes just 33 IGHV genes (table S5), the larger B-IGH locus encodes 99 germline IGHVs (table S6). There was a notable difference in the percentage of IGHV pseudogenes in the A-IGH (15%) locus compared to the B-IGH locus (46.5%), correlated with the size of the loci (Fig. 1, B and F). Both loci contain functional genes from the three major human IGHV clans but differ in the proportions of genes from each of the seven human V_H_ families (Fig. 1, C, G, and I). We observe expansion of IGHV3 functional genes in B-IGH compared to A-IGH. Proportionally, A-IGH is enriched for functional IGHV1 family genes, while B-IGH contains the only functional IGHV7 and IGHV6 family genes. These differences are consistent with a model in which the full ancestral IGH locus was duplicated, followed by separate gene retention, losses, and duplications at each locus. Examination of productive rearrangements across six individuals revealed that inferred germline V-gene repertoires of A-IGH were largely invariant (25 of 29 functional genes expressed in all individuals), while B-IGH showed greater interindividual germline repertoire variation (34 to 46 of 53 annotated functional and ORF germline V genes expressed per individual) that was independent of sequencing depth (Spearman’s rho = −0.14, *P* = 0.80), with a hierarchical deletion pattern in two individuals suggesting potential structural haplotype variation at this locus (fig. S2, A and B). Together, these observations suggest that each locus is under different diversification and evolutionary pressures.

To further evaluate the evolutionary divergence and selection pressures acting on these loci, we investigated relatedness of the genes between and within loci using measures of phylogenetic diversity. Between loci, the IGHVs differed substantially (unweighted UniFrac distance = 0.85, i.e., 85% of evolution is locus-specific), with greater diversity in the V genes on B-IGH than those on A-IGH (phylogenetic diversity _A-IGH_ = 1.82; phylogenetic diversity _B-IGH_ = 4.42). Within A-IGH, IGHV genes were phylogenetically clustered [mean pairwise distance (MPD) _observed_ = 0.33, MPD _expected_ = 0.37, rank = 7/100, *z* score = −1.84], suggesting that the locus is under purifying selection. In contrast, B-IGH V genes were less related than expected by chance (MPD _observed_ = 0.4, MPD _expected_ = 0.37, rank = 94/100, *z* score = 1.46), suggesting that this locus is under diversifying selection or undergoing an adaptive radiation. Collectively, these data suggest different evolutionary histories resulting from different selective pressures. This could be a result of different functional roles in the immune response for cells with BCRs derived from A-IGH compared to B-IGH.

**Fig. 2.**
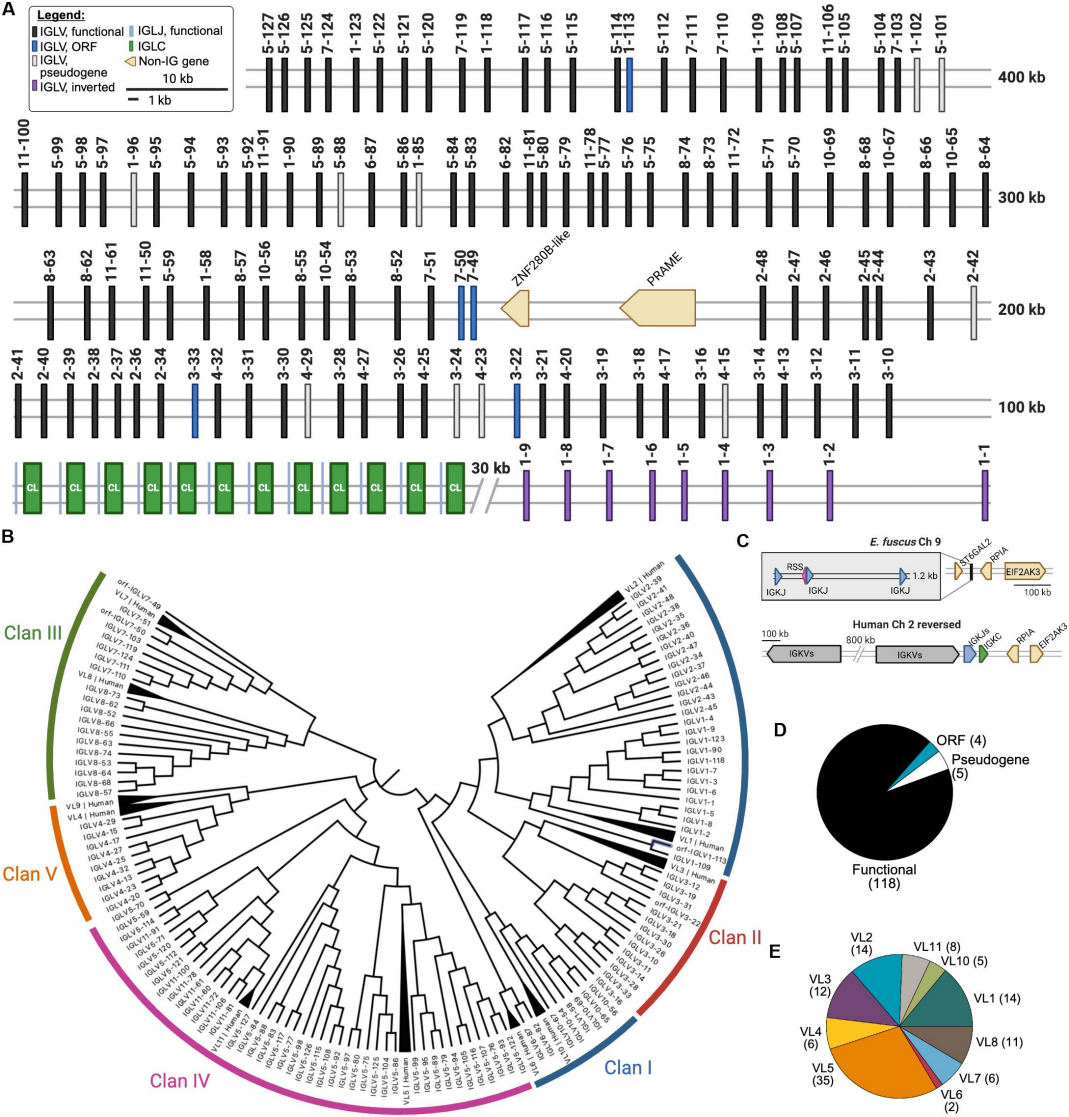
*E. fuscus* has a single Ig lambda light chain locus. (**A**) Schematic of IG gene organization for lambda locus contained on contig NC_072495.1 (**B**) Schematic of vestigial kappa locus on chromosome 9 with the human locus for comparison. (**C**) Pie chart of functionality and (**D**) family distribution for lambda variable genes. (**E**) Nearest neighbor-joining tree of functional and ORF V genes with human VL genes for reference and human clans. Created in BioRender. Pursell, T. (2026) https://BioRender.com/z7dpc6h.

Considering the IGH duplication, we asked whether *E. fuscus* has two light chain loci, as do other mammals and fish, or a single light chain locus, like birds. We identified a single light chain lambda locus (Fig. 2 and tables S7 to S9). The *E. fuscus* lambda locus on chromosome 23 (contig NC_072495.1) spans only 506 kb despite encoding 127 variable (Vλ; table S7), 12 joining (Jλ; table S8), and 12 constant (Cλ; table S9) genes. The locus is compact compared to the human kappa locus that encodes 116 genes but spans 1.9 Mb. The organization of Jλ and Cλ genes mirrors other species such that each Cλ gene is preceded by one Jλ gene, forming 12 J-C gene clusters (Fig. 2A). Each Jλ gene is flanked on the 5′ end by an RSS with a 12-base spacer and the Vλ genes with a 3′ RSS with a 23-base spacer as is observed in other species. Notably, the locus encodes nine functional Vλ genes downstream of the Jλ-Cλ gene cassettes in an inverted orientation (Fig. 2A). This organization is also seen in the equine lambda locus (*22*).

We found germline Vλ genes from all five human clans and all Vλ gene families except Vλ9 in *E. fuscus* (Fig. 2, B and E, and table S7). Of the 127 Vλ genes, 118 are functional, 4 are ORFs, and 5 are pseudogenes (Fig. 2D), nearly double the number of functional genes found in the human lambda and kappa light chain loci combined. In analyzing the rearranged repertoire, all functional Vλ genes, three ORFs, and a pseudogene, IGLV1-102P, were identified in productive rearrangements. Sequences using the inverted Vλ genes comprised 6% of the total repertoire, indicating that functional rearrangement does occur despite the noncanonical orientation.

Neither the NCBI eukaryotic genome annotation pipeline (*23*), nor IgDetective (*24*), nor initial BLAST (*25*) searching detected any kappa variable or constant-like genes in the current assembly. A focused search revealed only three IGKJ-like genes, only one of which has an RSS (Fig. 2C), suggesting loss of the IGK locus in the evolutionary history of *E. fuscus*. Additionally, we searched single-cell transcriptomic data presented below for evidence of a second light chain but found IgL in 89.8% of B lymphocytes and 96.7% of the cells for which we have complete, productive IGH sequence, with no evidence of kappa light chains. Together, these data support that *E. fuscus* has no functional or expressed kappa light chains, consistent with previous work (*26*).

**Fig. 3.**
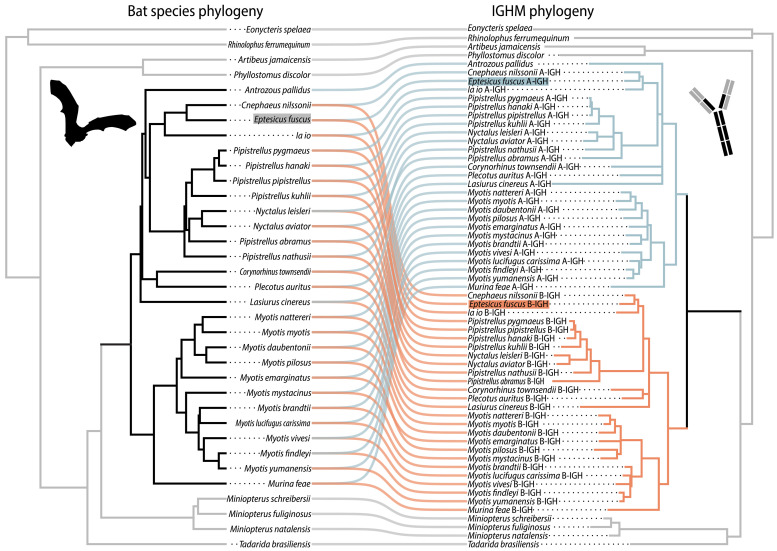
IGHM duplication phylogeny mirrors species phylogeny. The phylogenetic hypothesis on the left is the inferred relationship between the species, and the phylogenetic hypothesis to the right is the inferred relationships between IGHM loci; nodes with less than 75% bootstrap support were collapsed. Lines connect the species to its IGHM sequences. Non-vespertilionid branches are represented in gray. Within the Vespertilionidae, blue lines indicate A-IGH–like loci and orange lines indicate B-IGH–like loci. Our model species, *E. fuscus*, is highlighted in gray on the left and blue and orange on the right.

To understand the evolutionary origins of the duplication, we interrogated the publicly available genomes of 34 species of bats, including 26 closely related bats for evidence of IGH duplication, and found that the duplication is present in every vespertilionid we examined except *Antrozous pallidus* (Fig. 3 and table S10). On phylogenetic comparison, the IGHM sequences form two separate, well-supported clades by locus of origin (Fig. 3). Phylogenetic relationships within each IGHM clade parallel known species phylogenetic relationships (Fig. 3), supporting a single duplication of the IGH locus in the Vespertilionidae family ancestor after it diverged from Miniopteridae (∼37.5 Ma ago [95% confidence interval: 47 to 33 Ma]) or very early in the family’s history before the split of Vespertilioninae and Myotinae {∼26 Ma ago [95% confidence interval: 30 to 18 Ma]; (*27*)}. Some salmonid fish have two IGH loci, each on a separate chromosome, resulting from ancestral whole-genome tetraploidization (*28*). In general, teleost fish have undergone many genome duplications and losses, resulting in a diversity of IGH locus configurations (*28*). In contrast, no other mammals have two IGH loci and bats have no such history of whole-genome duplication. Bats do show duplication in other important immune genes (*29*, *30*), suggesting a separate, convergent evolutionary trajectory driven by selection on immunity.

Having determined that numerous vespertilionid bats have dual heavy chain loci, we sought to answer key questions about the use of these loci, specifically: (i) does allelic exclusion prevent individual B cells from making two different heavy chains; (ii) do interlocus V-D-J rearrangements occur; (iii) how do rearranged VDJ products differ between loci; (iv) is locus usage correlated with cell phenotype; (v) does class switching or SHM differ between the two loci; and (vi) is there evidence of selection favoring particular IGHV genes in antigen-experienced cells?

**Fig. 4.**
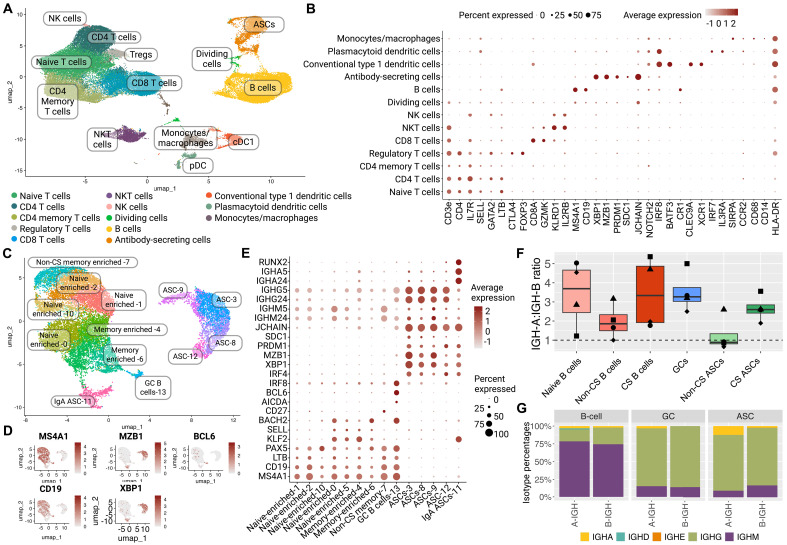
Splenic single-cell transcriptomics reveal distinct B cell phenotypes. (**A**) UMAP representation of single-cell transcriptomes from *E. fuscus* bats’ spleens (*n* = 4). (**B**) Dot plot of the main marker genes used to define each cell type. Dot size is proportional to the percentage of cells with detectable expression of the indicated gene, and dot color is indicative of the average expression value for the indicated gene, scaled across all identified clusters. (**C**) UMAP representation of subclusters within the B lymphocyte compartment. (**D**) Feature plot of log-normalized expression of marker genes. (**E**) Dot plot of the canonical B lymphocyte lineage and activation marker genes used for phenotype classification. (**F**) Box plot of ratio of A-IGH:B-IGH for each cell phenotype. Line represents the mean and ratio for each individual (*n* = 4) plotted with different shapes. Dashed line at ratio of 1. (**G**) Stacked bar plot of the percent of each isotype of BCRs from A-IGH and B-IGH for B cells, GC, and ASCs. Created in BioRender. Pursell, T. (2026) https://BioRender.com/vjn6qz8.

To address these questions, we first defined B cell and plasma cell phenotypes in *E. fuscus* using single-cell RNA sequencing (scRNAseq) on cryopreserved splenocytes from four individuals. After preprocessing, a total of 62,747 splenocytes were analyzed. Using canonical transcriptional markers, we identified clusters corresponding to major immune cell types (Fig. 4A). We identified two B lymphocyte lineage clusters, one enriched for B cell markers (i.e., *MS4A1*, encoding CD20, and *CD19*) associated with naive and memory phenotype cells, while the other was enriched for plasma cell markers (i.e., *XBP1*, *MZB1*, *PRDM1*, and *JCHAIN*) (Fig. 4B).

In humans, there are well-defined subsets of B cells found in the spleen (*31*) that have relatively similar counterparts in mice (*32*), but these are less well understood in other mammals. Previous studies have identified B lymphocytes in scRNAseq data in various bat species (*33*–*35*), but functional B cell subsets have not been fully defined, in part due to a lack of Ig gene annotations. Therefore, we undertook a focused analysis of B lymphocyte lineage subsets in *E. fuscus*. We identified a cluster analogous to germinal center (GC) cells that express canonical human markers including *BCL6*, *BACH2*, and *AICDA* (Fig. 4, C to E). Naive, transitional, and memory B cells showed less distinct cluster separation. This is consistent with other single-cell studies in various bat species (*33*–*35*), in which clusters were mixed or defined by noncanonical genes of unknown cell phenotype specificity. We found clusters enriched for memory-associated genes [e.g., *NOTCH2* (*36*), *ZBTB32* (*37*), and *CD27*], and others enriched (i.e., ≥50%) for B cells with low somatic hypermutation (SHM) frequencies (<1.5%), and expression of IGHM/IGHD isotypes presumed to be naive B cells (fig. S3).

Further genome annotation curation and experimental validation will be needed to fully define the gene expression patterns of naive and memory B cell populations, but we adopted a functional classification of naive B cells as those expressing low mutation IGHM or IGHD (SHM <1.5%). We grouped the remaining somatically mutated cells into non–class-switched B cells (non-csB cells) or class-switched B cells (csB cells) based on their expression of IGHM/D or other isotypes, respectively.

In contrast, plasma cell phenotype clusters were relatively homogeneous with elevated expression of canonical markers (i.e., *MZB1*, *JCHAIN*, and *XBP1*) except for a cluster of IgA class-switched plasma cells. This cluster is enriched for *RUNX2*, a transcription factor shown to induce IgA class switching in mature B cells during BCR signaling (*38*–*41*). Therefore, we divided the plasma cells into class-switched antibody-secreting cells (csASCs) and non–class-switched antibody-secreting cells (non-csASCs).

Allelic exclusion is the process that ensures that each B cell sequentially rearranges and expresses only one heavy and one light chain sequence despite having two chromosomal copies of each locus and, in many species, two different light chain loci. We hypothesized that allelic exclusion in bats with dual IGH loci would ensure that only a single IGH locus would be functionally rearranged to give an in-frame expressed protein product in each B lymphocyte. We assembled IGH rearrangements with CDR-H3s for 9922 (68%) of the 14,618 B lymphocytes identified in the gene expression dataset. After removing low-confidence assemblies, we found only 198 cells appearing to have two expressed in-frame CDR-H3 transcripts, or 2.0%. Given artifacts of single-cell sequencing, including the difficulty of completely excluding doublets, we interpret these findings as most consistent with allelic exclusion acting at the dual IGH loci.

To assess whether one of the two IGH loci is consistently rearranged first in the formation of the naive B cell repertoire, analogous to the ordered rearrangement of light chains that creates a kappa bias in human light chains, we assembled full-length IGH sequences from 5010 single B lymphocytes. If B cell precursor IGH V-D-J rearrangement produces a functional in-frame product one-third of the time, sequential attempts to rearrange both copies of A-IGH followed by B-IGH would result in a ratio of A-IGH to B-IGH usage in naive cells of approximately 2.25, whereas random selection of loci to rearrange first would result in a ratio of 1. The mean ratio of A-IGH to B-IGH locus usage observed in naive cells across the individuals is 3.41 (Fig. 4F). This bias supports a model of ordered rearrangement and allelic exclusion such that A-IGH rearrangements are attempted first, and only if no productive V-D-J rearrangement is obtained is rearrangement attempted at B-IGH, resulting in a larger percentage of naive cells with A-IGH BCRs compared to B-IGH. Other possible explanations for this ratio of A-IGH to B-IGH could include strong negative selection against developing B cells expressing the B-IGH locus, as could occur if such BCRs had high rates of self-reactivity (*42*).

We assessed whether VDJ rearrangement occurs between loci on different chromosomes by looking for agreement between the chromosomal origin of the V gene and the J genes. Over 95% of sequences had a V gene and a J gene from the same locus. We found similar patterns and no evidence of cross-loci rearrangement in the sequenced bulk BCR repertoires. Although we cannot fully rule out the possibility of rare interlocus recombination, it is likely that the apparent locus mismatches are due to errors in germline gene calling related to the high homology between certain V_H_, D, and J_H_ genes between the loci (fig. S1), and our limited knowledge of allelic variation between *E. fuscus* individual animals. We therefore used the V-gene locus alignment to define the origin of each IGH sequence in subsequent analysis.

To assess selection pressures affecting A-IGH or B-IGH usage in different B cell populations, we calculated the A-IGH:B-IGH ratio for each B cell subset for each individual bat (*n* = 4). Although the mean ratio for all cell subsets was greater than one, supporting an A-IGH bias across cell phenotypes, we observed the lowest mean ratio in non-csB cells and non-csASCs (mean ratio of 1.97 ± 0.90 and 1.25 ± 0.90, respectively) (Fig. 4F). A chi-square test comparing the frequency of A-IGH and B-IGH usage across the different B cell subsets showed a highly significant difference (*P* < 0.001), indicating a strong association between B cell phenotype and locus usage, strongly favoring the use of B-IGH locus BCRs in differentiated B lineage cells that have not undergone class switching.

We hypothesized that rearranged VDJs from different IGH loci would exhibit distinct V-gene segment usage in IgM-expressing B cells compared to IgG-expressing B cells, reflecting selection during antigen stimulation, as has been reported in human BCR repertoires (*43*, *44*). To capture a representative sample, expressed Ig rearrangements were enriched for IgM and IgG isotypes using 5′RACE (rapid amplification of cDNA ends) cDNA from flash-frozen splenic tissue of six animals, yielding a total of 307,312 productive BCR sequences. Genes from the VH3 family were the most common in both the IgM and IgG repertoires for both loci (fig. S4, A and B). However, for A-IGH, VH3 family gene usage significantly decreased in IgG compared to IgM (*P* < 0.05 by clone-wise nonpaired Wilcoxon rank-sum test), while VH1 family gene usage significantly increased (*P* < 0.05 by clone-wise nonpaired Wilcoxon rank-sum test). For B-IGH, there was almost no VH1 usage and heavy usage of VH7 family genes that significantly increased in IgG compared to IgM (*P* < 0.05 by clone-wise nonpaired Wilcoxon rank-sum test). These data demonstrate differences in the V-gene usage during generation of the naive repertoire for each IGH locus. Furthermore, differences in V-gene usage between IgM and IgG are consistent with locus-specific, antigen-driven selection.

To further investigate locus-specific effects of antigen experience on V-gene usage, we leveraged single-cell paired phenotypic and BCR sequence data. In naive B cells, IGHV3-2 accounted for the greatest proportion of expressed V genes (mean of 23.3% of naive B cells) across all bats in A-IGH–expressing naive cells, while B-IGH naive usage was more distributed across V3 family members, with IGHV3-94 being the most frequent (16.7%; fig. S5A). Consistent with antigen-driven selection, several V genes—including IGHV1-20 and IGHV3-30 from A-IGH, and IGHV6-2 from B-IGH—were significantly enriched in class-switched memory B cells and ASCs relative to naive B cells [false discovery rate (FDR) < 0.05; fig. S5B]. Fewer genes were significantly enriched in the B-IGH–expressing cells, which could be a result of similar V-gene usage in naive cells compared to other B cell subsets or, more likely, a result of the shallow sampling of B-IGH cells.

Antibody functions such as complement activation and antibody-dependent cellular cytotoxicity (ADCC) are mediated by the constant region or isotype of an antibody. To investigate the possibility of different functional roles for cells using a particular locus, we assessed rates of class switching within each B cell subset. Notably, IgA class-switched B cells and ASCs disproportionately used the A-IGH locus (median of 38 IgA-expressing cells from A-IGH locus and 7 cells from B-IGH; a complete summary of absolute cell numbers in table S11), indicating that this locus may have particular importance for mucosal humoral responses (Fig. 4G). Decreased class switching to IgA in the B-IGH locus could be related to the unusual, oversized switch region between IGHG and IGHE compared to A-IGH (Fig. 1G), although IgE-expressing cells were rare overall. Additionally, ASCs using B-IGH were more likely to be non–class-switched (i.e., expressing IgM; mean ratio of 1.25 ± 0.90 for non–class-switched ASCs compared to mean ratio of 2.66 ± 0.68 for class-switched ASCs) compared to those using A-IGH (Fig. 4, F and G). Therefore, despite the greater diversity of IGHV genes in the B-IGH locus, it is more often used in non–class-switched cells. Although we do not know the distribution of anatomical niches in which these cells may have spent time, non–class-switched cells are described in other mammals as being part of primary and extrafollicular humoral responses (*45*–*47*). Overall, these data support a model in which the B-IGH locus is less efficient at class switching, potentially due to decreased recruitment or accessibility of AID, or that B cells using the B-IGH locus are less likely to enter immunological niches promoting class switching, such as the GC.

To further evaluate functional differences between the two IGH loci, we measured the frequency of SHM in VDJ rearrangements. SHM is mediated by focused recruitment of the AID enzyme to the Ig loci and is the basis for affinity maturation in which B cells expressing higher affinity for antigen preferentially expand within the stimulated clone. Rates of SHM ranged up to 22.7%. As expected, we observed the lowest mean SHM in IgM sequences (1.8%) and higher mean rates in IgA and IgG (4.8% and 4.1%, respectively) in the B cell clusters. The SHM rates were not significantly different in ASCs compared to non-naive B cells for all isotypes except IgM (*P* < 0.05 by clone-wise nonpaired Wilcoxon rank-sum test).

**Fig. 5.**
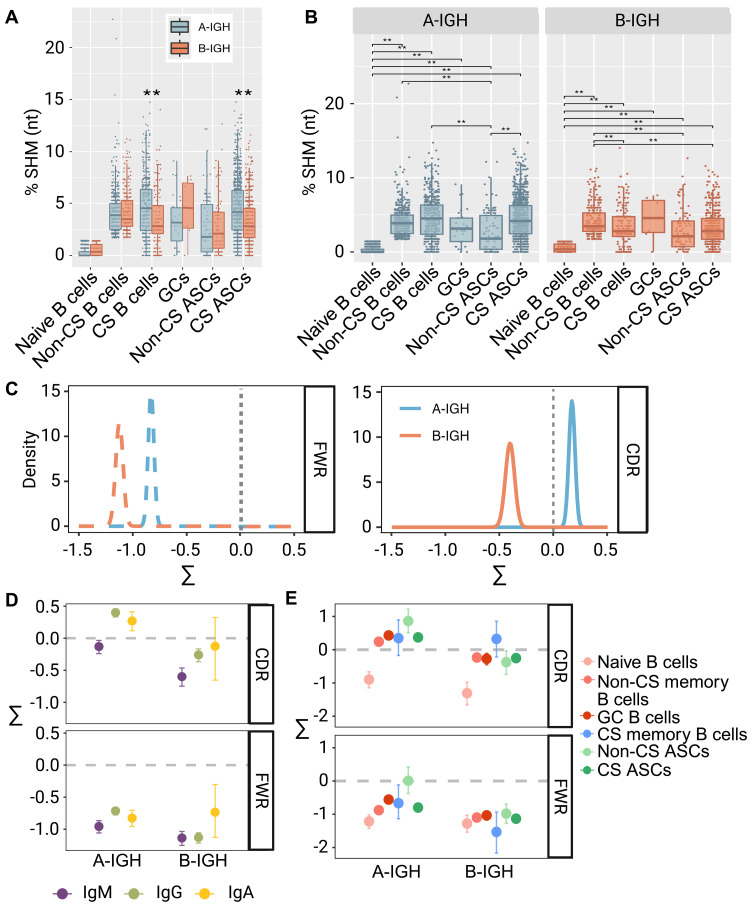
Levels of SHM vary between and within IGH loci. (**A**) Distribution of percent nucleotide SHM of IGHV segment for A-IGH (blue) and B-IGH (orange) clones grouped by cell phenotype. (**B**) Distribution of percent nucleotide SHM of IGHV segment for each clone, grouped by phenotype and locus. (**C**) Density plot of selection scores (sigma, 𝝨) of the CDRs (solid line, top panel) and FWR (dashed line, bottom panel) for IGHV segments from A-IGH (blue) and B-IGH (orange) clones. Threshold for positive selection gray dashed line (𝝨 = 0). (**D**) Plot of mean with confidence interval for the selection scores of CDR (top panel) and FWR (bottom panel) grouped by locus for IgM (purple), IgG (green), and IgA (yellow). (**E**) Plot of mean with confidence interval for selection scores of CDR (top panel) and FWR (bottom panel) grouped by locus for each cell phenotype. Comparisons between groups were performed with the Wilcoxon rank-sum test. **P < 0.05. Graphs generated in R and figure created in BioRender. Pursell, T. (2026) https://BioRender.com/n7qe2mh.

We observed a strikingly higher SHM frequency in csB cells and csASCs using A-IGH sequences compared to B-IGH (Fig. 5A and fig. S6; *P* < 0.001 by clone-wise nonpaired Wilcoxon rank-sum test for each subset). In contrast, the lower levels of SHM in non-csB cells, non-csASCs, and GC cells did not differ between loci (Fig. 5A and fig. S6). These results, in combination with the higher proportion of non-csASCs using the B-IGH locus, suggest a model in which the access or activity of AID at the B-IGH locus is lower than at the A-IGH locus.

Among B lineage cells using the A-IGH locus, csASCs had higher levels of SHM compared to non-csASCs (*P* < 0.001, clone-wise Kruskal-Wallis rank-sum test followed by Dunn’s multiple comparisons), while csB cells did not differ in SHM from non-csB cells (Fig. 5B, left panel). This supports a similar mechanism as seen in humans that results in class switching early in activation and maintenance of low mutation rates in IgM. In contrast, for B cells and ASCs using B-IGH, non-csB cells had higher SHM than csB cells, with no difference in SHM of csASCs and non-csASCs (Fig. 5B, right panel). We hypothesize that B cells using B-IGH remain non–class-switched through multiple rounds of SHM, but that once class switching has occurred, the recruitment of AID to the B-IGH locus for SHM is impaired.

An alternative explanation for reduced SHM in B-IGH could be decreased density of AID hotspots. Preferential targeting of cytosines within WRC motifs (where W = A/T and R = A/G) by AID means that AGY serine codons (AGC/AGT) are themselves WRC hotspot motifs, while TCN codons are not. Thus, V genes with higher AGY serine codon frequency are effectively pre-enriched for AID targeting sequences predicted to result in higher SHM rates independent of chromatin accessibility or transcriptional regulation. To explore this hypothesis as a potential explanation for differences in SHM between loci, we first assessed AID hotspot and serine codon frequencies across germline V genes in the A-IGH and B-IGH loci. When compared to closest human homologous germline V genes (fig. S7A), *E. fuscus* germline V genes have significantly lower AGY serine codon content (fig. S7, B and D) and AID hotspot (fig. S7, C and E) density in CDR1 and CDR2 compared to their closest human homologs (both *P* < 0.001 by Wilcoxon). This is consistent with the overall lower rates of SHM in the *E. fuscus* repertoires compared to human repertoires. Contrary to the hypothesis that B-IGH has reduced intrinsic AID targeting, B-IGH germline genes show no significant difference in AID hotspot density (fig. S7D) compared to A-IGH in all regions examined. This suggests that intrinsic germline AID hotspot frequencies are unlikely to account for the observed SHM differences between loci. Since the difference in SHM was only observed in class-switched memory B cells (csmemB cells) and ASC (csASCs), we compared the intrinsic AID hotspot density of germline genes to the mean observed SHM for both B cell subsets in IgG class-switched cells (fig. S7, F and G). We observe weak negative correlation or lack of correlation between germline AID hotspot frequency and SHM frequencies. Together, these data raise the possibility that locus-specific SHM differences are more likely due to factors that could include chromatin accessibility, locus architecture, or transcriptional regulation rather than germline AID target frequencies.

Antigen experience shapes the BCR repertoire through selective expansion of B cells with high-affinity receptors. Positive selection for improved antigen binding occurs mainly in the complementary-determining regions (CDRs) of an antibody, while negative selection against destabilizing mutations acts most strongly on the intervening structural framework regions (FWRs). Selection scores (sigma, 𝝨) representing the log odds ratio (OR) of observed replacement-to-silent mutation frequency compared to the expected frequency are higher in CDRs compared to the FWRs for both A-IGH and B-IGH (Fig. 5C). However, positive selection (𝝨 > 0) only reached significance for the CDRs of A-IGH, but not B-IGH (Fig. 5C). This is likely due to the overall lower levels of SHM in B-IGH sequences limiting detection of positive selection above background. This evidence of positive selection is only found in IgG and IgA, but not IgM, for A-IGH sequences (Fig. 5D).

## DISCUSSION

In summary, while *E. fuscus* bats share many common mechanisms of humoral immunity with other mammals such as allelic exclusion, SHM, and class switching, this analysis of high-quality long-read chromosome-level assemblies for *E. fuscus* and other vespertilionid bats identified a previously unknown IGH locus duplication in mammals, highlighting the extreme immunogenetic variability of these complex, repetitive loci in bats. These data underscore the importance of genomic investigation of more species across the chiropteran order to better understand their true immunological diversity. These data also suggest that previous serological studies of infection or vaccination in bats may have overlooked some aspects of bat antibody responses. Future infection studies in bats could include comprehensive serological profiling that measures IgM, IgG, and IgA responses, as current methods focused on IgG alone may not adequately capture the full breadth of responses. In addition, reagents should be validated to ensure that they measure antibodies encoded by A-IGH and B-IGH loci in bats having this duplication. Previous work has established that *E. fuscus* can mount robust antibody responses to vaccination and infection, but these can be transient, as seen with neutralizing titers to rabies virus (*48*–*51*). Neutralizing assays usually do not discriminate between antibody isotypes, and it is possible that a portion of these responses in *E. fuscus* may be attributable to rapid, short-lived IgM derived from non–class-switched memory cells and non–class-switched ASCs potentially dominated by B-IGH–expressing cells. Recent work has also highlighted the importance of IgM in early innate-like antibody responses and complement activation in other mammalian systems (*52*–*54*).

Our data in *E. fuscus* also prompt the hypothesis that antibodies derived from B-IGH with its higher gene segment diversity but lower class switching and SHM frequencies could provide broader but lower-affinity antigen recognition, complemented by A-IGH–derived antibodies that have higher SHM frequencies, greater evidence of affinity maturation selection, and higher rates of class switching to IgG or IgA for distinct effector functions. Additional investigation of antigen-specific B cell responses to defined infections or vaccinations will provide further tests of the validity of this model of bat humoral immunity. The presence of the dual IGH loci in these bats also provides a unique internally controlled opportunity to understand the relationships between BCR locus sequences and their regulatory regions, as well as other mechanisms of B cell development and functional immunity.

## MATERIALS AND METHODS

### Animals

This study was carried out in accordance with recommendations set forth in the National Institutes of Health *Guide for the Care and Use of Laboratory Animals* (*55*). Big brown bats (*E. fuscus*) used for this study were collected from a single colony in Calhoun (GA, USA). Collection of animals occurred under Georgia Department of Natural Resources permit #29-WSF-16-189. Capture, handling, and experimental procedures were performed in compliance with requirements of Centers for Disease Control and Prevention (CDC) Institutional Animal Care and Use Committee–approved protocol 2809ELLBATC.

### *E. fuscus* Ig locus annotations

The *E. fuscus* DD_ASM_mEF_20220401 assembly and non-Ig gene annotations generated with the NCBI Eukaryotic Genome Annotation Pipeline from the NCBI database (*23*) were used to retrieve the contigs containing the Ig heavy and light sequences (or *RPIA* and *EIF2AK3* in the case of the IGK locus). IgDetective (*24*) (v1.1.0) was used to identify contigs containing Ig heavy and light chain loci as well to predict putative V, J, and D genes.

Additionally, variable, joining, and constant genes for heavy, lambda, and kappa loci from all species within the international ImMunoGeneTics information system (IMGT) database (*56*) (v1.2.9) were mapped onto corresponding contigs using Geneious Prime 2021.1.1. Candidate gene segments were categorized as functional, ORF, and pseudogenes according to “Functionality” of IMGT (*56*). Leader sequences were manually annotated. RSSs were identified and filtered based on previously described recombination information content (RIC) score thresholds (*57*, *58*). We classified V-gene units as pseudogenes or ORFs based on IMGT criteria (*56*). Last, V-gene annotations were validated and additional genes were identified by alignment of bulk IgM and IgG repertoires from 5′RACE (details below).

### AID hotspot density

We used an interval spanning from the most 3′ IGHJ gene to 5 kb after the transmembrane domain of IGHA to search in both strands for the occurrence of the AID hotspot motif 5′-AGCT-3′ (*59*) using DNA-Pattern in RSA tools (*60*). Raw counts were estimated in 1-kb nonoverlapping windows, graphs were generated in R (v4.3.1) (*61*), and figures were created using BioRender.com.

### Comparative IGHV and IGLV phylogenetic analysis

Functional, representative germline heavy and lambda variable genes from the major families for humans were collected from IMGT (*62*). Multiple sequence alignment was performed on the *E. fuscus* and human IGHVs with MUSCLE, and a nearest neighbor-joining tree was generated with Jukes-Cantor using Geneious Prime 2021.1.1 (https://www.geneious.com). The final figure was generated using Fig.Tree (v1.4.4). For IGLV, the approach was identical. IGHV sequences from A-IGH (*n* = 28) and B-IGH 24 (*n* = 53) were aligned using DECIPHER (*63*), and phylogenetic distances were calculated using the Jukes-Cantor model with pairwise deletion. A neighbor-joining tree was constructed using the ape package (*64*). Unweighted and weighted UniFrac distances were calculated using the phyloseq R package (v1.46.0) (*65*). MPD was calculated as the average phylogenetic distance between all sequence pairs within each locus using the picante package (*66*). Statistical significance of MPD values was assessed using standardized effect sizes comparing observed values to null distributions generated with 99 randomizations under the “richness” null model, where negative standardized effect sizes indicate phylogenetic clustering and positive values indicate overdispersion.

### Species phylogeny and comparative IGHM alignment

To investigate the evolutionary origin of the IGH duplication, we examined publicly available bat genomes with a focus on vespertilionids with high-quality genomes (table S10). We located and extracted chromosomes and/or scaffolds containing regions encoding IgH constant genes using genome annotations and/or by applying BLAST (*25*) with IGHC genes from other species. We initially annotated exons by mapping IGHC genes from other species to the regions and aligned the IGHM region in Geneious Prime 2021.1.1 (https://www.geneious.com) and corrected it manually. The final alignment spans from the beginning of the CH1 to near the end of the M domain. The phylogenetic hypothesis for the IGHM genes was generated using PhyML with a GTR+R substitution scheme, which was the best supported model (*67*, *68*) and was run with 100 bootstrap replicates. To determine whether the evolutionary history of the IGH loci mirrors the evolutionary history of the bat species, we plotted the consensus IGHM phylogenetic hypothesis with a published species phylogenetic hypothesis (*27*) using the “cophylo” command in phytools (*69*). *Myotis vivesi*, which is not present in the mammal-wide phylogenetic hypothesis, was added manually based on its relationship to other taxa (*70*). Any nodes in the IGHM phylogenetic hypothesis with less than 75% support were collapsed to a polytomy in the figure. The final alignment and consensus phylogeny are available as files S1 and S2, respectively.

### Calculation of expected A-IGH:B-IGH ratio

We calculated the expected ratio of A-IGH:B-IGH cells in naive populations under the assumption that recombination occurs in an ordered fashion starting at A-IGH as follows. Successful recombination assumed to occur one-third of the time such that productive A-IGH: 1/3 (allele #1A) + 2/3 * 1/3 (allele #2A) yields 0.56 for A-IGH and productive B-IGH: 2/3 * 2/3 * 1/3 (allele #1B) + 2/3 * 2/3 * 2/3 * 1/3 (allele #2B) yields 0.25. Therefore, the A-IGH:B-IGH ratio is 0.56:0.25 or 2.24 for mature naive cells.

### A-IGH:B-IGH ratio

Within each group (i.e., naive and non–class-switched B cells), sequences were grouped by bat. Then, the number of cells with BCRs from A-IGH plus a pseudo-count of one was divided by the number of cells with BCRs from B-IGH plus a pseudo-count of one. Frequency of A-IGH versus B-IGH for cumulative data was compared with a chi-square test.

### Bulk Ig repertoire methods

RNA was isolated from flash-frozen *E. fuscus* splenic tissue using Zymogen quick DNA/RNA mini kit (catalog no. D7001), and 5′RACE cDNA was generated using SMARTer 3′/5′RACE kit (Takara, catalog no. 634858). Nested constant region primers for IGHM, IGHG, and IGLCs (table S12) were generated from known sequences and chromosome-level assembly annotations generated above. A touchdown polymerase chain reaction (PCR) protocol using Takara SeqAmp DNA polymerase was used for the first round PCR as follows: (i) 5 cycles of 94°C for 30 s and 72°C for 3 min; (ii) 5 cycles of 94°C for 30 s, 70°C for 30 s, and 72°C for 3 min; and (iii) 25 cycles of 94°C for 30 s, 68°C for 30 s, and 72°C for 3 min. The PCR product from round 1 was diluted in Tris-EDTA buffer and used as a template for a second round of three-step PCR using the superFi II DNA polymerase (Thermo Fisher Scientific, catalog #12351010) with an initial denaturation of 98°C for 30 s, then 25 cycles of 98°C for 10 s, 60°C for 30 s, and 72°C for 30 s, and final extension of 5 min. A dual-sided SPRIselect (Beckman Coulter, catalog no. B23317) bead size selection was performed on PCR products. Libraries were barcoded using NEBNext DNA library kit (New England Biolabs, #E7645S and #E7370L). Libraries were pooled and sequenced using a MiSeq Reagent Kit v3 600 cycle (Illumina, catalog no. MS-102-3003) on an Illumina MiSeq instrument. Reads were merged and trimmed using FLASH and aligned to the genome using Geneious Prime 2021.1.1 to validate annotations and identify additional genes. Data were then processed using MiXCR (*71*) (v4.6.0) with a custom reference annotation of BCRs and used to generate inferred allelic germline reference sets for each individual animal to be used below for single-cell analysis. Full allele-level sequences and quality metrics as well as single-cell validation results are provided in the Supplementary Materials (S3_IGH_Allele_Evaluation.xls).

### Single-cell transcriptomic data generation

Single-cell suspensions were generated from a portion of fresh whole spleens and cryopreserved in complete medium with 10% dimethyl sulfoxide (DMSO). Cells were thawed, washed once, counted, and resuspended in a 1× phosphate-buffered saline (PBS) + 0.2% bovine serum albumin (BSA). Approximately 20,000 cells from a single bat were loaded per 10× lane. The Chromium GEM Single Cell 5′ Kit v2 (10x Genomics, PN-1000374) was used, and protocol Rev D was followed for the preparation of cDNA and gene expression library generation.

For BCR enrichment libraries, nested constant region primers were generated from known sequences and chromosome-level assembly annotations (table S12). Custom outer constant region reverse primers were pooled and added (0.2 μM final) to 50 μl of Amp Mix (10x Genomics, catalog no. 2000047), forward primer (IDT based on 10x Genomics published sequence, 0.2 μM), 2 μl of cDNA, and water. Thermocycler with lid set to 105°C was cycled as follows: (i) 98°C for 45 s; (ii) 98°C for 20 s, 55°C for 30 s, 72°C for 1 min for 8 to 12 cycles; (iii) 72°C for 1 min; and (iv) hold at 4°C. After double-sided bead selection, 35 μl of sample was added to pooled custom inner constant region reverse primers (0.2 μM final), 50 μl of Amp Mix, forward primer (IDT based on 10x Genomics published sequence, 0.2 μM), and water. Thermocycler with lid set to 105°C was cycled as follows: (i) 98°C for 45 s; (ii) 98°C for 20 s, 62°C for 30 s, 72°C for 1 min for 8 to 12 cycles; (iii) 72°C for 1 min; and (iv) hold at 4°C. The final amplified products were processed, and libraries were prepared and barcoded as detailed in the 10x Genomics protocol Rev D. After library preparation, quality control was performed using a bioanalyzer (Agilent 2100 Bioanalyzer, Agilent Technologies) and quantification, pooling, and dilution of libraries to 1.5 nM were performed using KAPA library quantification kit (Roche, catalog no. 07960140001). Gene expression libraries were pooled and sequenced using a Nova-seq SP 200 cycle kit (Illumina, San Diego, CA; catalog no. 20040719) on an Illumina NovaSeq instrument. Gene expression and BCR libraries were pooled and sequenced using a Nova-seq S2, 200 cycle kit (catalog no. 20028315) on an Illumina NovaSeq instrument. All raw sequencing reads have been deposited in NCBI’s Short Read Archive (BioProject PRJNA1425580). The code used in this study is available at https://doi.org/10.5281/zenodo.18764310.

### Single-cell transcriptomic data analysis

A custom cellranger reference was generated using the DD_ASM_mEF_20220401 assembly and annotations available on NCBI. Annotations for IG genes were removed and replaced with custom annotations. Single-cell gene expression data were processed using Cell Ranger (10x Genomics, v.8.0.0) followed by Seurat (*72*) (v5.0.1). Quality control measures were first applied to filter out cells with an unusually high or low number of detected genes, indicative of potential cell stress or death. Normalization of the data was performed using the NormalizeData function to mitigate the influence of cell-specific biases. The FindVariableFeatures function identified highly variable genes across the dataset, which were used for downstream analysis.

Before initial clustering, IG genes were removed from the highly variable list. Datasets were integrated using canonical correlation analysis (CCA), and layers were joined. Clustering was repeated, and the dead cell cluster was removed. Principal components analysis (PCA) was conducted using the RunPCA function on the scaled data to reduce dimensionality. The significant principal components were selected based on the Elbow plot, guiding the selection of dimensions for clustering. Cell clusters were identified using the FindClusters function. The RunUMAP function was then used to visualize the cells in a two-dimensional uniform manifold approximation and projection (UMAP) plot. Differential expression analysis between identified cell clusters was performed using the FindAllMarkers function with a Wilcoxon rank-sum test. Marker genes were used to annotate clusters based on known cell type–specific expression profiles. Low-quality cells were discriminated by distinct clustering, expression of only mitochondrial and ribosomal genes, and lack of other phenotypic gene expression.

To identify B lymphocyte subsets, B lymphocyte lineage clusters and dividing cells were reclustered; light chain genes were removed from the variable gene set, but isotype genes were included. Cells were clustered, and non-B cells and doublets formed distinct clusters and were removed. Remaining cells were reclustered to yield the final subsets.

### Single-cell BCR data analysis

Paired-end reads generated by single-cell BCR sequencing were assembled into contigs representing full-length V(D)J recombinations for IGH and CDR-3 for IGL using MiXCR (*71*) (v4.6.0). Individual germline references containing putative alleles generated from bulk IG data (above) were used to process the data for each individual. The number of nucleotide changes per sequence was determined and normalized to the length of the V-gene region to calculate the mutation frequency. For cell barcodes with multiple IGH contigs, a dominant contig was identified if one chain had (i) more than five times the reads of the other or (ii) three times more unique molecular identifiers (UMIs) and the isotype for the dominant chain agreed with the transcriptomic expression for that cell. For IGH assemblies without an isotype, gene expression data from the single-cell transcriptomes were leveraged to identify the isotype. An isotype was called in cases where only a single constant gene has nonzero count or if the same constant gene from both loci have nonzero counts (e.g., both A-IGHG and B-IGHG). Assemblies were then filtered to only include those that were identified as B lineage cells in the transcriptomic analysis.

### Selection pressure analysis

Single-cell IGH BCR sequences with germline reference were exported in the IMGT format using MiXCR (*71*) (v4.6.0). Sequences were filtered for gaps, and then SHazaM (*73*–*77*) (v1.2.0) BASELINe was applied for analysis of selection pressure. Visualizations were generated in R using SHazaM (v1.2.0) and then processed with Biorender.com.

### Germline V-gene sequence identity analysis

*E. fuscus* germline IGHV gene sequences were aligned against the IMGT human germline reference database using IgBLAST. For each *E. fuscus* germline V-gene sequence, the closest human homolog and percent nucleotide identity were extracted from an Adaptive Immune Receptor Repertoire (AIRR)-formatted tabular output. V-gene sequences were grouped by human V-gene family (e.g., IGHV1 and IGHV3). Results are presented as a dot plot showing percent identity to the closest human homolog for each *E. fuscus* germline V gene, with the mean identity per gene indicated by a vertical bar.

### Germline V-gene AID hotspot and serine codon analysis

The AGY serine codon frequency and AID hotspot density were calculated for each germline V-gene sequence across V-gene regions. AGY serine codon frequency was defined as the proportion of total codons consisting of AGC or AGT, which are AID-targetable WRC motifs. AID hotspot density was calculated as the number of WRC/AGY motif occurrences (matching the pattern AGC|AGT|AGCT|[AT][AG]C|[AT][AG]T) per codon. The same metrics were computed for the matched homologous human germline V-gene sequences. Mean values per region were summarized in heatmap. Differences in AGY and AID hotspot frequency between loci were assessed using the Kruskal-Wallis test, and differences between *E. fuscus* and human homologs were assessed using the Wilcoxon rank-sum test.

### Germline V-gene AID content and observed SHM

Single-cell BCR sequences from IGHG class-switched memory B cells and class-switched antibody-secreting cells were grouped by germline V gene and locus, and mean observed SHM was calculated. The relationship between germline gene AID hotspot frequency and mean observed SHM was assessed by linear regression per locus. Point size was scaled to V-gene usage frequency within each cell population. Two linear mixed-effects models were fit using the lme4 package in R. Both models used log-transformed SHM as the outcome and included individual and V gene as random intercepts to account for repeated measures within individuals and systematic differences in mutability between V genes. Model 1 included locus (A-IGH versus B-IGH) as the sole fixed effect. Model 2 additionally included scaled germline AID hotspot frequency as a covariate. Models were compared by likelihood ratio test. Analyses were performed separately for class-switched memory B cells and class-switched antibody-secreting cells.

### V-gene enrichment analysis across B cell differentiation states

A binary expression matrix was constructed from single-cell BCR sequencing data, scoring each cell-gene pair as expressed (1) or not (0) for each V gene. Analyses were performed separately for each locus. V-gene segments were excluded if expressed in fewer than 10 cells total, if any cell type had zero expressing cells, or if any cell type had fewer than two expressing cells. For each V-gene segment, a generalized linear mixed-effects model (GLMER) was fit using lme4 in R. ORs and 95% confidence intervals were estimated using broom.mixed::tidy. *P* values were corrected for multiple comparisons using the Benjamini-Hochberg FDR procedure (significance threshold: FDR < 0.05). Results were visualized as a forest plot in ggplot2.

### Statistical analysis

All statistical analyses were conducted in R. All comparisons are clone-wise for bulk IG data where a clone is defined as a unique sequence or cell-wise for single-cell data unless otherwise stated. Nonpaired Wilcoxon rank-sum tests were used to conduct pairwise comparisons. Multiple comparisons were conducted using Kruskal-Wallis rank-sum tests followed by Dunn’s multiple test with Benjamini-Yekutieli for FDR correction. Permutation tests were performed in python with the DABEST (*78*) package using 5000 permutations of unpaired Cliff’s delta between the groups. The 95% confidence interval was bias-corrected and accelerated. Statistical significance markers were added to graphs using ggpubr (*79*) package or manually added during figure generation with Biorender.com.
