# Supplementary material for: Immunoglobulin heavy chain locus duplication in bats
Source: Sci Adv. 2026 Jul 29;12(31):eaeb6714. doi: 10.1126/sciadv.aeb6714 (PMC13418733; doi:10.1126/sciadv.aeb6714)
Supplement: Supplementary file 1 — Figs. S1 to S7 Tables S1 to S12 Legends for data S1 to S3 [file sciadv.aeb6714_sm.pdf]

Science Advances
AAAS

Supplementary Materials for

Immunoglobulin heavy chain locus duplication in bats

Taylor Pursell *et al.*

Corresponding author: Scott D. Boyd, publications_scott_boyd@stanford.edu;
Hannah K. Frank, hkfrank@tulane.edu

Sci. Adv. **12**, eaeb6714 (2026)
DOI: 10.1126/sciadv.aeb6714

The PDF file includes:

Figs. S1 to S7
Tables S1 to S12
Legends for data S1 to S3

Other Supplementary Material for this manuscript includes the following:

Data S1 to S3

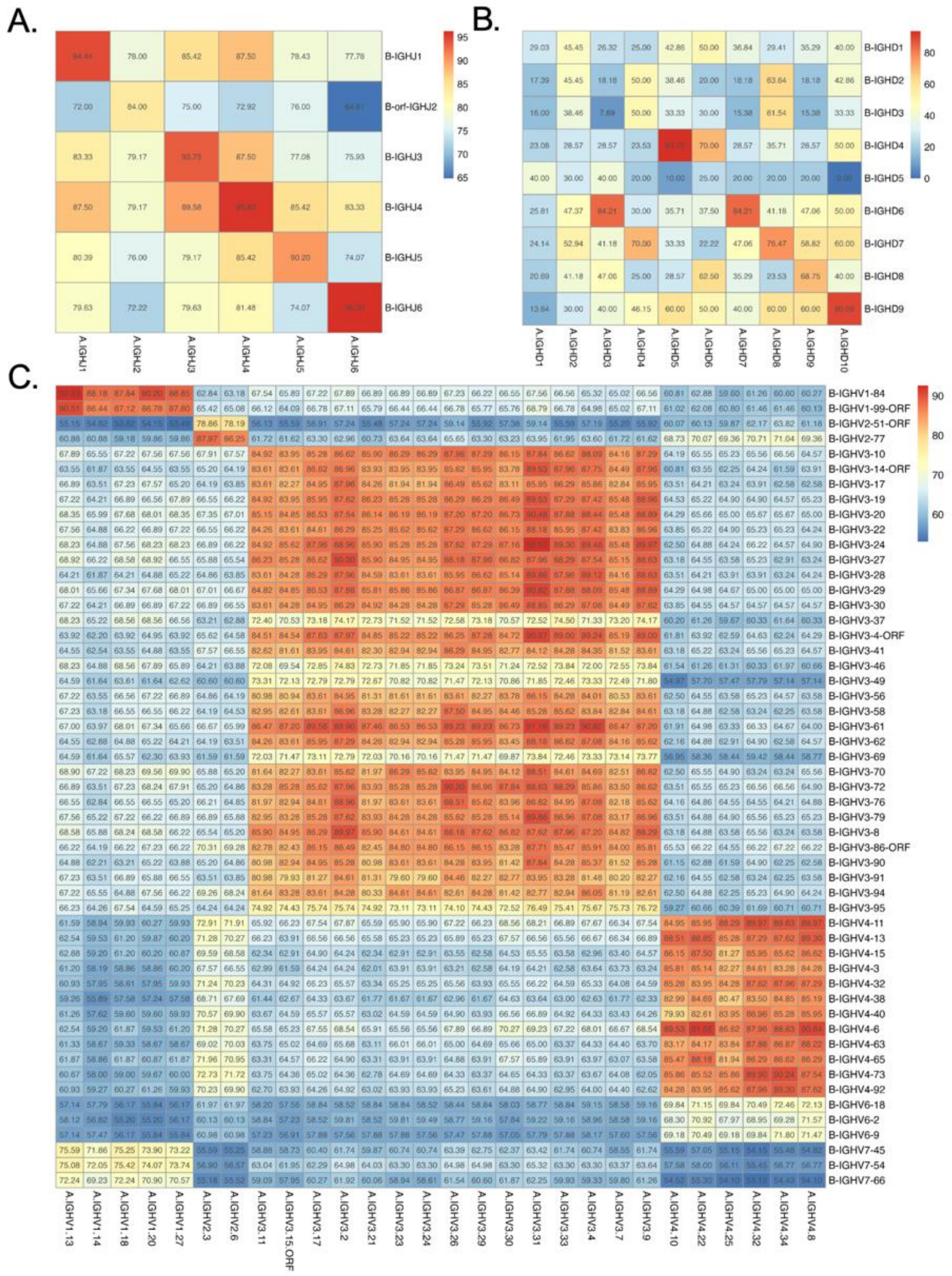

Fig. S1. Cross locus IG gene homology. Heatmaps of the distance matrix of (**A**) joining genes, (**B**) diversity, and (**C**) variable genes from A-IGH compared to B-IGH where the color and number indicate the percent nucleotide identity for the given pair of V genes. MUSCLE alignment with iterations (n = 10) clustered with neighbor joining and CLUSTALW sequence weighting scheme. Created in BioRender. Pursell, T. (2026) https://BioRender.com/wnug12x.

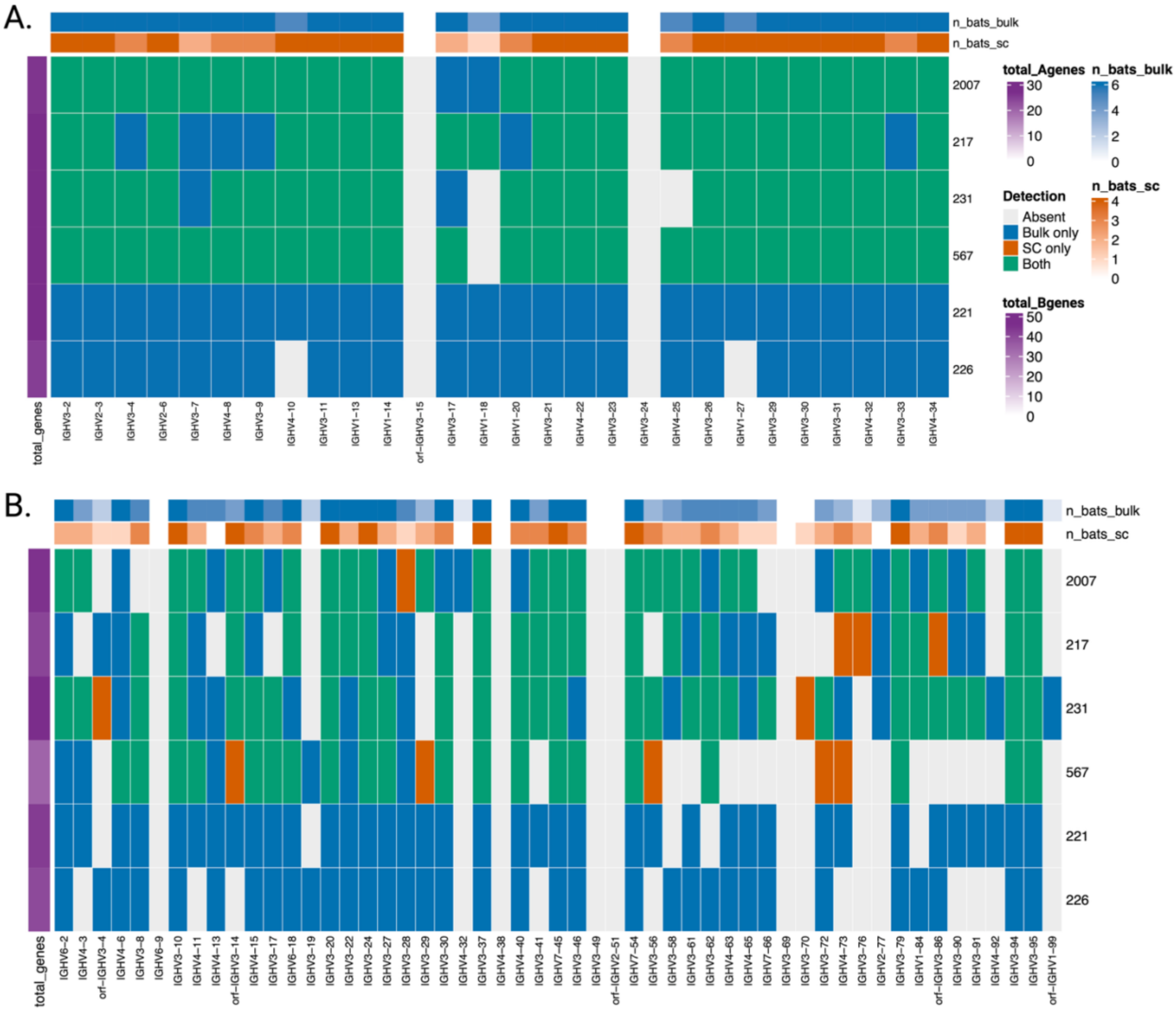

Fig. S2 Variation in inferred germline V gene repertoires across individuals and datasets. Heatmaps showing the expression of **(A)** A-IGH and **(B)** B-IGH functional and ORF variable genes (columns) across *Eptesicus fuscus* individuals (rows) detected in bulk 5' RACE (n=6) and/or single cell (n=4) BCR sequencing. Blue indicates the gene was detected bulk BCR only, orange in single cell only, green in both; white indicates germline gene was absence from both datasets. Column annotation (top) indicates the total number of bats in which the gene was detected in either bulk BCR (blue gradient) or single cell (orange gradient), and row annotation (left) indicates the number of V-genes expressed by individual (purple gradient). Columns are grouped such that those with both single cell and bulk data (n=4) are on top and those with only bulk on the bottom (n=2)). Rows are not clustered to preserve genomic order. Figure created in BioRender. Pursell, T. (2026) https://BioRender.com/ukaw5mf.

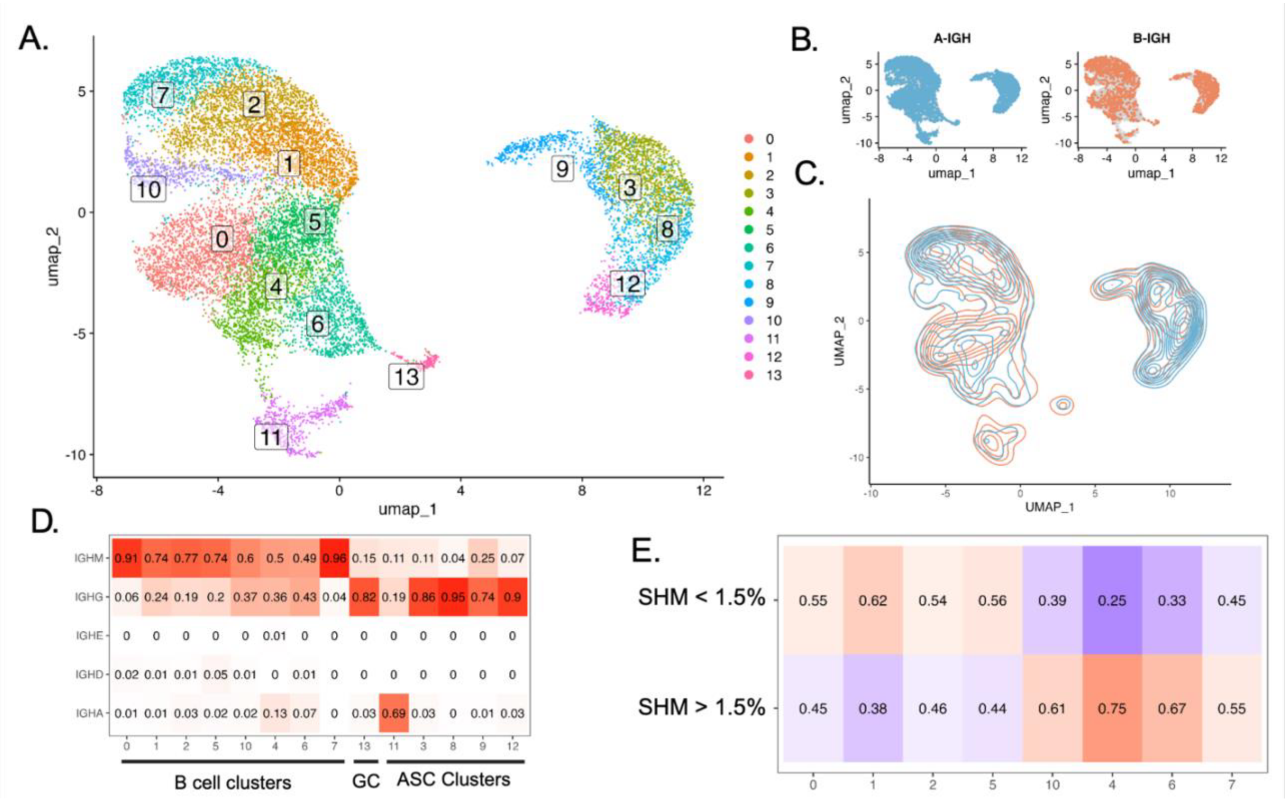

Fig. S3. Identifying B cell subsets in spleen data. (A) UMAP of B lymphocyte lineage subclusters. **(B)** Dot plot of cells expressing A-IGH, left and B-IGH right. **(C)** Density plot of A-IGH expressing cells (blue) and B-IGH expressing cells (orange). **(D)** Heatmap of proportion of cells expressing each isotype within each subcluster; square color intensity and label based on the proportion. **(E)** Heatmap of proportion of IGHM expressing cells with low SHM (<1.5%) compared to high SHM (>1.5%) within the B cell subclusters; square color intensity and label based on the proportion. Created in BioRender. Pursell, T. (2026) https://BioRender.com/8yzmae1

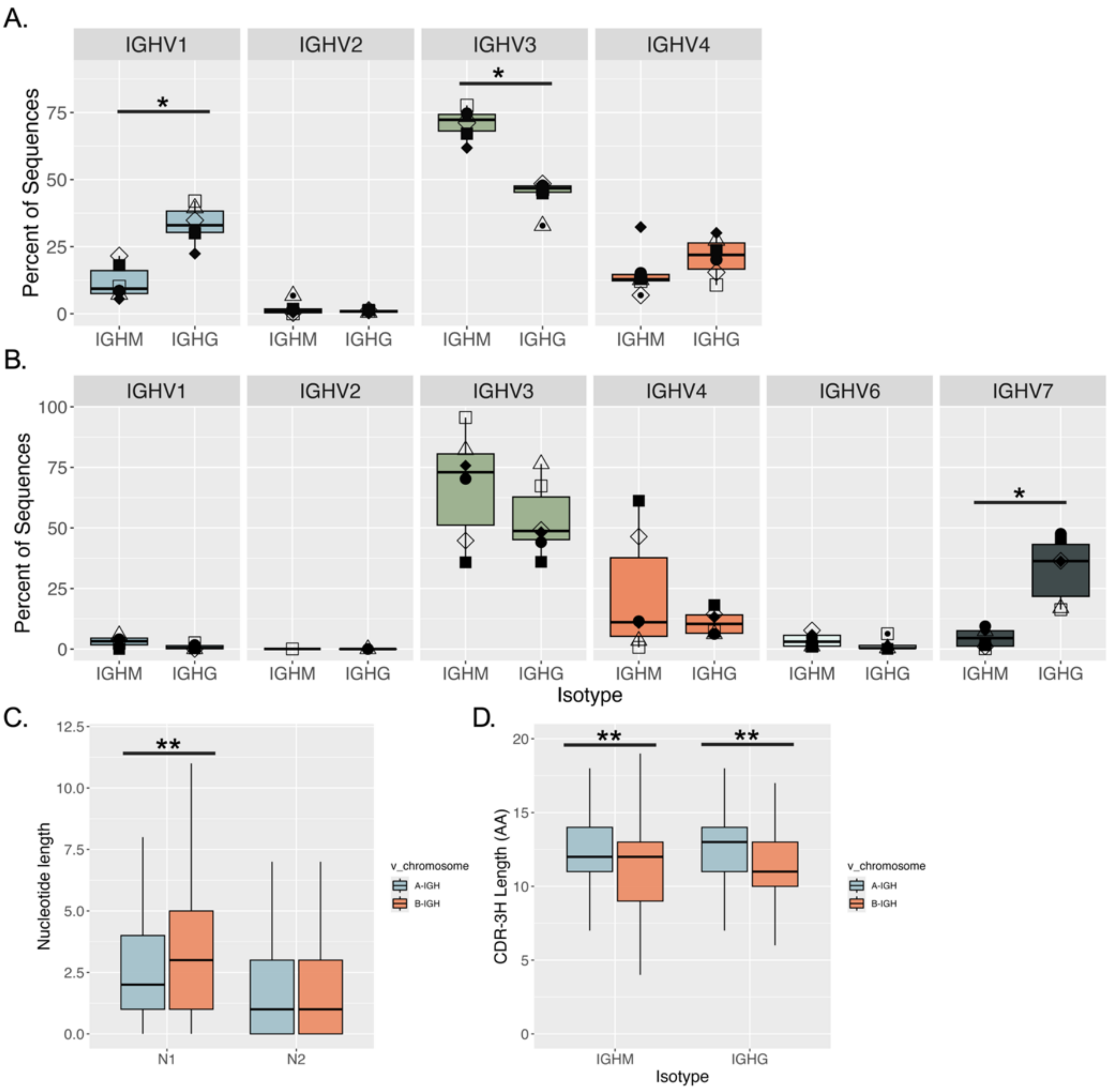

Fig. S4. Analysis of bulk BCR repertoires from spleen. VH-family percentages for **(A)** A-IGH and **(B**) B-IGH within IGHM and IGHG from productive rearrangements found in bulk repertoire data where each point represents an individual bat (n = 6). **(C)** Box-and-whisker plot of length of V-D (N1 nucleotides) and D-J junctions (N2 nucleotides) for IGHM for A-IGH (blue) and B-IGH (orange). The horizontal line represents the median and whiskers span the range of the data excluding the outliers **(D)** Box-and-whisker plot of CDR-3H amino acid lengths including cysteine and tryptophan for IGHM and IGHG for A-IGH (blue) and B-IGH (orange). The horizontal line represents the median and whiskers span the range of the data excluding the outliers. *p < 0.005,**p < 0.000001, defined by clone wise unpaired Wilcoxon rank sum test. Graphs generated in R. Post-processing and figures created in BioRender. Pursell, T. (2026) https://BioRender.com/zeaq39p.

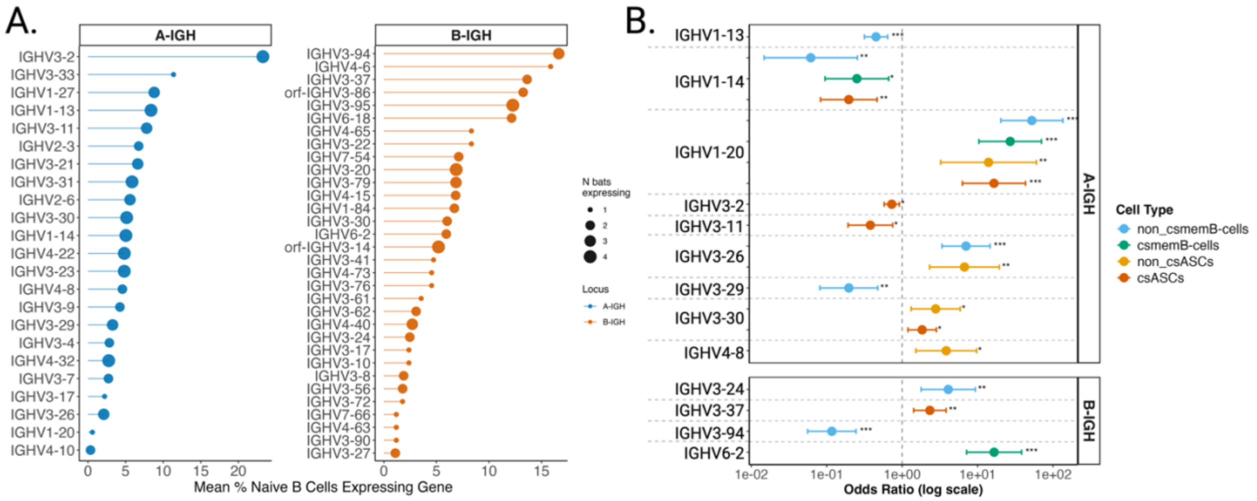

Fig. S5. V gene usage frequency varies across B cell subsets between IGH loci. **(A)** V gene usage in A-IGH (**left** panel, blue) and B-IGH (**right** panel, orange) expressing naive B cells. Each point represents a unique V gene, with point size indicating the number of individuals (n = 4) in which the gene was detected, and position on the x-axis indicating the mean percentage of naive B cells expressing that gene across individuals. Genes with usage exceeding 5% are labelled. **(B)** Enrichment of V gene usage in A-IGH (**left** panel) and B-IGH (**right** panel) expressing cells across B cell subsets relative to naive B cells, estimated using logistic regression. Each point represents a significant V gene–cell type association (FDR < 0.05, Benjamini-Hochberg correction applied separately within each locus). Odds ratios > 1 indicate enrichment relative to naive B cells; odds ratios < 1 indicate depletion. Error bars represent 95% confidence intervals. Circle = A-IGH locus; triangle = B-IGH locus. Cell types are color-coded as indicated. Results are faceted by locus. Significance thresholds: * FDR < 0.05, ** FDR < 0.01, *** FDR < 0.001. Figure created in BioRender. Pursell, T. (2026) https://BioRender.com/8k5qmvd

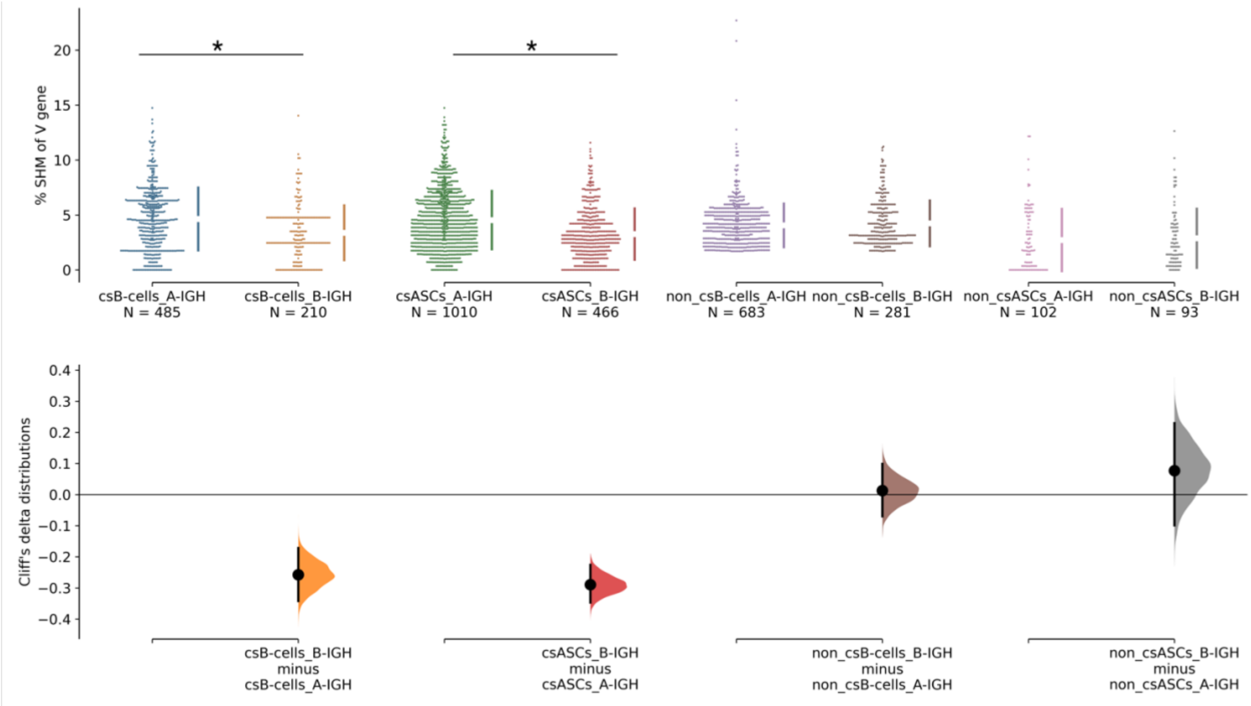

Fig. S6. Permutation analysis for SHM differences between loci by B cell subset. Top plot contains swarm plot of percent SHM of V gene sequences grouped by cell type comparing A-IGH (left) and B-IGH (right). Bottom plot is a main effect size plot non-paired Cliff's delta with its 95% confidence interval for permutations of the non-parametric comparisons between A-IGH and B-IGH for each cell type. * $p < 0.001$ Created in BioRender. Pursell, T. (2026) https://BioRender.com/gi50y0n.

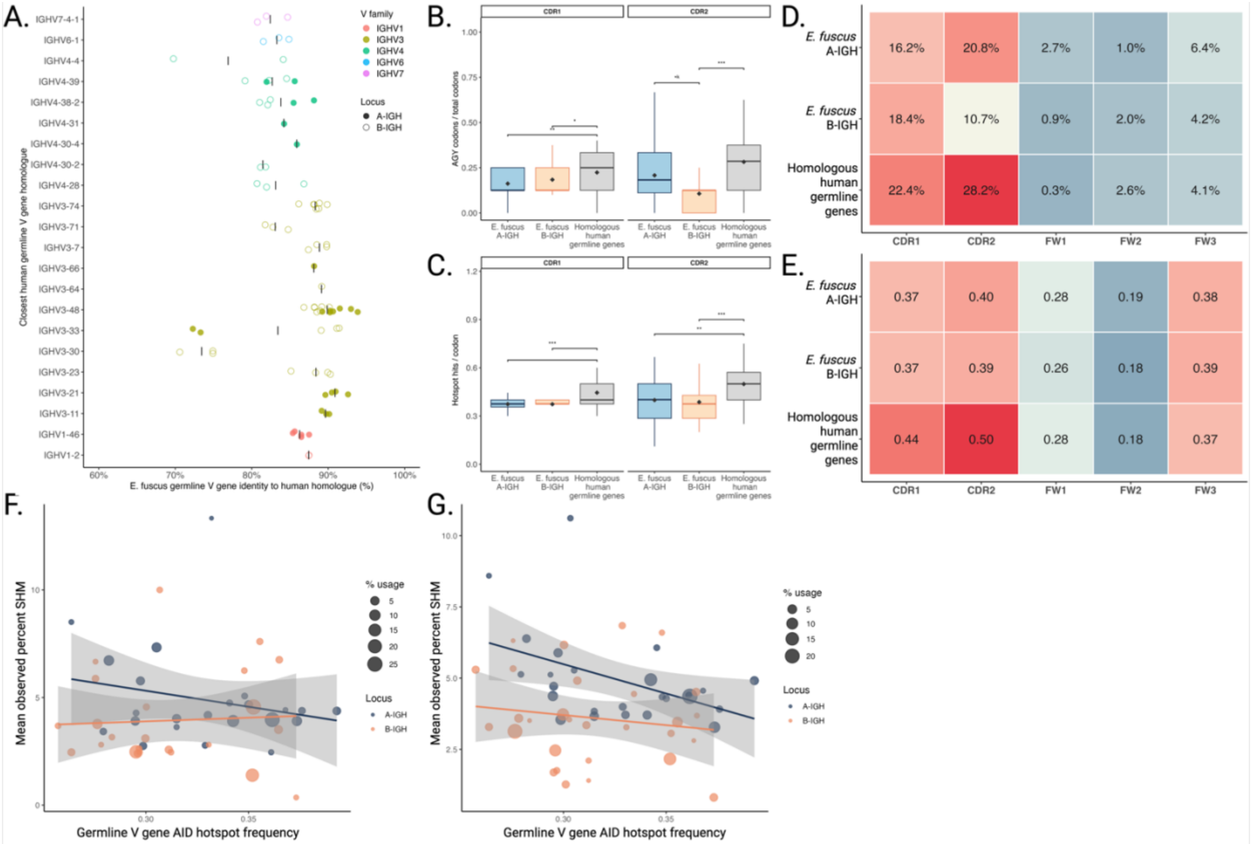

Fig. S7. Germline V gene sequence identity, intrinsic AID hotspot frequency, and relationship to observed somatic hypermutation. **(A)** Dot plot showing percent nucleotide identity of each *E. fuscus* germline V gene sequence to its closest human homologue, grouped by human germline V gene, V gene family (color), and locus of origin (filled circle = A-IGH; open circle = B-IGH). Vertical bar indicates mean identity per human germline homologue. **(B)** AGY serine codon fraction and **(C)** AID hotspot frequency (WRC/AGY motif hits per codon) across V gene regions (CDR1 and CDR2) for *E. fuscus* A-IGH (blue), *E. fuscus* B-IGH (orange), and homologous human germline genes (grey). Diamond indicates mean. Significance brackets indicate pairwise comparisons between groups; A-IGH vs B-IGH was tested by Kruskal-Wallis, all other comparisons by Wilcoxon rank-sum test (* $p<0.05$, ** $p<0.01$, *** $p<0.001$; non-significant comparisons not shown). **(D)** Heatmap of mean AGY serine codon percent and **(E)** mean AID hotspot frequency per region for *E. fuscus* A-IGH, B-IGH, and homologous human germline genes. **(F-G)** Scatter plots of mean observed SHM per V gene versus intrinsic germline AID hotspot frequency for IGHG class-switched cells from **(F)** class-switched memory B cells and **(G)** class-switched antibody-secreting cells. Each point represents one V gene, sized by usage frequency within that cell population. Lines indicate linear trends per locus (A-IGH = blue, B-IGH = orange) with 95% confidence intervals. Figure created in BioRender. Pursell, T. (2026) https://BioRender.com/u18oj83.

Table S1. IGHC gene annotations for A-IGH

Locus	Designation	Exon	Genome Position [a]
A-IGH	A-IGHM	CH1	254914 - 255232
		CH2	255317 - 255652
		CH3	255914 - 256228
		CH4	256572 - 256904
		CH-S	256905 - 256966
		TM1	259152 - 259267
		TM2	259353 - 259361
A-IGH	A-IGHD	CH1	269606 - 269870
		H1	270195 - 270284
		H2	271683 - 271765
		CH3	271766 - 272099
		TM1	274229 - 274389
		TM2	274390 - 274395
A-IGH	A-IGHG	CH1	296810 - 297095
		H1	297371 - 297415
		CH2	297507 - 297836
		CH3	297926 - 298246
		CH-S	298247 - 298254
		TM1	299615 - 299745
		TM2	300352 - 300435
A-IGH	A-IGHE	CH1	320077 - 320383
		CH2	320502 - 320822
		CH3	320907 - 321227
		CH4	321310 - 321636
		CH-S	321637 - 321644
		TM1	324629 - 324730
		TM2	324851 – 324958
A-IGH	A-IGHA	CH1	333612 - 333918
		H1	334117 - 334157
		CH2	334158 - 334464
		CH3	334653 - 334985
		CH-S	334986 - 335047
		TM	338349 – 338578

[a] Contig NC_072477.1

Table S2. IGHC gene annotations for B-IGH

Locus	Designation	Exon	Genome Position [b]
B-IGH	B-IGHM	CH1	1784216 - 1784534
		CH2	1784607 - 1784945
		CH3	1785177 - 1785491
		CH4	1785820 - 1786152
		CH-S	1786153 - 1786214
		TM1	1788792 - 1788907
		TM2	1789001 - 1789009
B-IGH	B-IGHD	CH1	1801850 - 1802113
		H1	1802396 - 1802482
		H2	1803897 - 1803979
		CH3	1803980 - 1804313
		TM1	1806140 - 1806288
		TM2	1806289 - 1806295
B-IGH	B-IGHG	CH1	1818229 - 1818514
		H1	1818752 - 1818793
		CH2	1818888 - 1819217
		CH3	1819307 - 1819627
		CH-S	1819628 - 1819635
		TM1	1820925 - 1821055
		TM2	1829396 - 1829479
B-IGH	B-IGHE	CH1	1859123 - 1859429
		CH2	1859567 - 1859887
		CH3	1859954 - 1860292
		CH4	1860370 - 1860699
		CH-S	1860700 - 1860707
		TM1	1864143 - 1864276
		TM2	1864623 - 1864730
B-IGH	B-IGHA	CH1	1873005 - 1873308
		H1	1873478 - 1873500
		CH2	1873501 - 1873807
		CH3	1873998 - 1874330
		CH-S	1874331 - 1874392
		TM	1876670 - 1876899

[b] Contig NC_072496.1

Table S3. IGHJ gene annotations for A-IGH and B-IGH

Locus	Designation	Expressed	Genome Position
A-IGH	A-IGHJ1	Y [c,d]	247052 – 247105[a]
A-IGH	A-IGHJ2	Y [c,d]	247365 - 247414 [a]
A-IGH	A-IGHJ3	Y [c,d]	247697 - 247744 [a]
A-IGH	A-IGHJ4	Y [c,d,e]	248015 - 248062 [a]
A-IGH	A-IGHJ5	Y [c,d]	248398 - 248448 [a]
A-IGH	A-IGHJ6	Y [c,d]	248930 - 248983 [a]
B-IGH	B-IGHJ1	Y [c,d]	1777158 - 1777211 [b]
B-IGH	B-IGHJ2[ORF]	Y [c]	1777808 - 1777855 [b]
B-IGH	B-IGHJ3	Y [c,d]	1777808 - 1777855 [b]
B-IGH	B-IGHJ4	Y [c,d]	1778083 - 1778130 [b]
B-IGH	B-IGHJ5	Y [c,d]	1778466 - 1778516 [b]
B-IGH	B-IGHJ6	Y [c,d]	1778884 - 1778937 [b]

[a] Contig NC_072477.1; [b] Contig NC_072496.1; [c] Functional rearranged in at least one individual in bulk IG repertoire; [d] Functional rearranged in $\geq$ 1 individual in single cell repertoire; [P] Pseudogene; [ORF] Open reading frame; [e] Putative allele found in $\geq$ 1 individual

Table S4. IGHD gene annotations for A-IGH and B-IGH

Locus	Designation	Length (nt)	Expressed [c,d]	Genome Position
A-IGH	A-IGHD1	31	Y	233577 - 233607 [a]
A-IGH	A-IGHD2	22	Y	233913 - 233934 [a]
A-IGH	A-IGHD3	19	Y	234857 - 234875 [a]
A-IGH	A-IGHD4	20	Y	235089 - 235108 [a]
A-IGH	A-IGHD5	16	Y	235511 - 235526 [a]
A-IGH	A-IGHD6	10	Y	236216 - 236225 [a]
A-IGH	A-IGHD7	19	Y	236580 - 236598 [a]
A-IGH	A-IGHD8	17	Y	236812 - 236828 [a]
A-IGH	A-IGHD9	16	Y	236984 - 236999 [a]
A-IGH	A-IGHD10	10	Y	246699 - 246708 [a]
B-IGH	B-IGHD1	33	Y	1763176 - 1763208 [b]
B-IGH	B-IGHD2	13	Y	1763773 - 1763785 [b]
B-IGH	B-IGHD3	15	Y	1763989 - 1764003 [b]
B-IGH	B-IGHD4	16	Y	1766304 - 1766319 [b]
B-IGH	B-IGHD5	10	Y	1767058 - 1767067 [b]
B-IGH	B-IGHD6	19	Y	1767404 - 1767422 [b]
B-IGH	B-IGHD7	18	Y	1767651 - 1767668 [b]
B-IGH	B-IGHD8	16	Y	1767830 - 1767845 [b]
B-IGH	B-IGHD9	10	Y	1776812 - 1776821 [b]

[a] Contig NC_072477.1; [b] Contig NC_072496.1; [c] Functional rearranged in $\geq$ 1 individual in bulk analysis; [d] Functional rearranged in $\geq$ 1 individual in single cell analysis [P] Pseudogene, [ORF] Open reading frame

Table S5. IGHV gene annotations for A-IGH

Family	Locus	Proposed Designation	Expressed	Allele(s)	Human homolog	Homolog identity (%)	Genome Position[a]
1	A-IGH	A-IGHV1-1[P]	--	--	--	--	231604 - 229122
1	A-IGH	A-IGHV1-5[P]	--	--	--	--	201160 - 196756
1	A-IGH	A-IGHV1-12[P]	--	--	--	--	158537 - 153960
1	A-IGH	A-IGHV1-13	Y[b,c]	Y[d]	IGHV1-46	87.5	153490 - 148227
1	A-IGH	A-IGHV1-14	Y[b,c]	Y	IGHV1-46	85.6	147757 - 145163
1	A-IGH	A-IGHV1-18	Y[b,c]	--	IGHV1-46	86.5	133495 - 129585
1	A-IGH	A-IGHV1-20	Y[b,c]	Y	IGHV1-46	85.4	126195 - 122974
1	A-IGH	A-IGHV1-27	Y[b,c]	Y	IGHV1-46	86.5	92354 - 92825
2	A-IGH	A-IGHV2-3	Y[b,c]	Y	IGHV3-33	73.3	212751 - 204865
2	A-IGH	A-IGHV2-6	Y[b,c]	Y	IGHV3-33	72.3	196285 - 190476
2	A-IGH	A-IGHV3-9	Y[b,c]	--	IGHV3-48	90.6	180693 - 170120
2	A-IGH	A-IGHV3-11	Y[b,c]	Y	IGHV3-11	89.1	163267 - 159007
3	A-IGH	A-IGHV3-2	Y[b,c]	Y	IGHV3-48	93.0	228634 - 213222
3	A-IGH	A-IGHV3-4	Y[b,c]	Y	IGHV3-48	93.9	204382 - 201627
3	A-IGH	A-IGHV3-7	Y[b,c]	--	IGHV3-11	89.7	189984 - 185742
3	A-IGH	A-IGHV3-9	Y[b,c]	Y	IGHV3-48	90.6	180693 – 170120
3	A-IGH	A-IGHV3-15[ORF]	Y[b]	--	IGHV3-48	89.2	144676 - 140485
3	A-IGH	A-IGHV3-16[P]	--	--	--	--	139991 - 137248
3	A-IGH	A-IGHV3-17	Y[b,c]	--	IGHV3-2	89.6	136760 - 133965
3	A-IGH	A-IGHV3-19[P]	--	--	--	--	129100 - 126665
3	A-IGH	A-IGHV3-21	Y[b,c]	Y	IGHV3-11	90.1	122480 - 118230
3	A-IGH	A-IGHV3-23	Y[b,c]	Y[d]	IGHV3-48	90.2	111260 - 107041
3	A-IGH	A-IGHV3-24	Y[a]	--	IGHV3-48	90.2	103834 - 104323
3	A-IGH	A-IGHV3-26	Y[b,c]	Y	IGHV3-21	90.5	96569 - 97054
3	A-IGH	A-IGHV3-29	Y[b,c]	--	IGHV3-21	92.6	79943 - 80431
3	A-IGH	A-IGHV3-30	Y[b,c]	Y	IGHV3-66	88.2	78023 - 78513
3	A-IGH	A-IGHV3-31	Y[b,c]	Y	IGHV3-48	91.5	74092 - 74577
3	A-IGH	A-IGHV3-33	Y[b,c]	Y	IGHV3-21	91.0	65288 - 65776
4	A-IGH	A-IGHV4-8	Y[b,c]	Y	IGHV4-30-4<br>IGHV4-31	85.9	185267 - 181181
4	A-IGH	A-IGHV4-10	Y[b,c]	Y	IGHV4-38-2	85.5	169649 - 163761
4	A-IGH	A-IGHV4-22	Y[b,c]	Y	IGHV4-38-2	88.1	117758 - 111749
4	A-IGH	A-IGHV4-25	Y[b,c]	--	IGHV4-39	81.6	99145 - 99617
4	A-IGH	A-IGHV4-28[P]	--	--	--	--	84996 - 85332
4	A-IGH	A-IGHV4-32	Y[b,c]	Y	IGHV4-31	84.2	70047 - 70523
4	A-IGH	A-IGHV4-34	Y[b,c]	Y	IGHV4-39	85.6	62260 - 62735

[a] Contig NC_072477.1, [b] Functional BCR in at least one individual in bulk analysis, [c] Functional BCR in at least one individual in single cell analysis, [P] Pseudogene, [ORF] open reading frame, [d] Putative allele found in $\geq$ 1 individual

Table S6. IGHV gene annotations for B-IGH

Family	Locus	Proposed designation	Expressed	Allele	Human homolog	Homolog identity (%)	Genome Position[a]
1	B-IGH	B-IGHV1-64 [P]	--	--	--	--	16979486 - 16979961
1	B-IGH	B-IGHV1-74 [P]	--	--	--	--	17057215 - 17057678
1	B-IGH	B-IGHV1-84	Y[b,c]	Y	IGHV1-2	87.5	17157598 - 17158077
1	B-IGH	B-IGHV1-88 [P]	--	--	--	--	17184987 - 17185458
1	B-IGH	B-IGHV1-96 [P]	--	--	--	--	17285813 - 17286389
1	B-IGH	B-IGHV1-99 [ORF]	Y[b]	--	IGHV1-2	87.5	17301061 - 17301498
2	B-IGH	B-IGHV2-51 [ORF]	N	--	IGHV3-30	70.6	16896960 - 16897380
2	B-IGH	B-IGHV2-77	Y[b]	--	IGHV4-4	69.8	17096111 - 17096580
3	B-IGH	B-IGHV3-1 [P]	--	--	--	--	16503414 - 16503816
3	B-IGH	B- IGHV3-4 [ORF]	Y[b,c]	--	IGHV3-48	88.2	16527789 - 16528195
3	B-IGH	B-IGHV3-7 [P]	--	--	--	--	16556888 - 16557291
3	B-IGH	B-IGHV3-8	Y[b,c]	Y	IGHV3-23	89.9	16560533 - 16560935
3	B-IGH	B-IGHV3-10	Y[b,c]	--	IGHV3-74	88.1	16570805 - 16571208
3	B-IGH	B-IGHV3-12 [P]	--	--	--	--	16582709 - 16583111
3	B-IGH	B-IGHV3-14 [ORF]	Y[b,c]	Y	IGHV3-48	88.2	16606837 - 16607244
3	B-IGH	B-IGHV3-17	Y[b,c]	--	IGHV3-23	88.4	16623445 - 16623848
3	B-IGH	B-IGHV3-19	Y[b]	--	IGHV3-7	88.5	16634141 - 16634544
3	B-IGH	B-IGHV3-20	Y[b,c]	Y[d]	IGHV3-7	89.8	16638301 - 16638701
3	B-IGH	B-IGHV3-22	Y[b,c]	Y	IGHV3-74	88.4	16650678 - 16651081
3	B-IGH	B-IGHV3-24	Y[b,c]	--	IGHV3-74	89.9	16665389 - 16665792
3	B-IGH	B-IGHV3-26 [P]	--	--	--	--	16681975 - 16682378
3	B-IGH	B-IGHV3-27	Y[b,c]	--	IGHV3-23	90.3	16685649 - 16686052
3	B-IGH	B-IGHV3-28	Y[b,c]	Y	IGHV3-74	88.9	16696254 - 16696657
3	B-IGH	B-IGHV3-29	Y[b,c]	--	IGHV3-7	89.8	16700373 - 16700773
3	B-IGH	B-IGHV3-30	Y[b,c]	--	IGHV3-7	87.4	16716588 - 16716990
3	B-IGH	B-IGHV3-33 [P]	--	--	--	--	16733309 - 16733715
3	B-IGH	B-IGHV3-36 [P]	--	--	--	--	16750812 - 16751226
3	B-IGH	B-IGHV3-37	Y[b,c]	Y	IGHV3-30	74.9	16764040 - 16764425
3	B-IGH	B-IGHV3-41	Y[b,c]	--	IGHV3-33	89.1	16810686 - 16811098
3	B-IGH	B-IGHV3-42 [P]	--	--	--	--	16831837 - 16832249
3	B-IGH	B-IGHV3-43 [P]	--	--	--	--	16833699 - 16833900
3	B-IGH	B-IGHV3-46	Y[b,c]	Y	IGHV3-30	74.9	16850124 - 16850509
3	B-IGH	B-IGHV3-48 [P]	--	--	--	--	16879081 - 16879082
3	B-IGH	B-IGHV3-49	Y[b]	--	IGHV3-71	81.8	16885573 - 16885973
3	B-IGH	B-IGHV3-50 [P]	--	--	--	--	16894044 - 16894455
3	B-IGH	B-IGHV3-55 [P]	--	--	--	--	16927207 - 16927588
3	B-IGH	B-IGHV3-56	Y[b,c]	--	IGHV3-74	86.1	16935682 - 16936094
3	B-IGH	B-IGHV3-58	Y[b,c]	--	IGHV3-64	89.2	16948156 - 16948563
3	B-IGH	B-IGHV3-59 [P]	--	--	--	--	16951922 - 16952367
3	B-IGH	B-IGHV3-61	Y[b,c]	--	IGHV3-48	90.1	16964700 - 16965184
3	B-IGH	B-IGHV3-62	Y[b,c]	--	IGHV3-48	89.2	16968603 - 16969101
3	B-IGH	B-IGHV3-67 [P]	--	--	--	--	17018077 - 17018615
3	B-IGH	B-IGHV3-68 [P]	--	--	--	--	17028332 - 17028823
3	B-IGH	B-IGHV3-69	N	--	IGHV3-71	82.8	17030286 - 17030768
3	B-IGH	B-IGHV3-70	Y[c]	--	IGHV3-74	88.8	17034814 - 17035309
3	B-IGH	B-IGHV3-72	Y[b,c]	--	IGHV3-48	89.0	17048349 - 17048845
3	B-IGH	B-IGHV3-75 [P]	--	--	--	--	17066902 - 17067385
3	B-IGH	B-IGHV3-76	Y[b,c]	--	IGHV3-48	88.5	17079119 - 17079611
3	B-IGH	B-IGHV3-79	Y[b,c]	--	IGHV3-74	88.2	17125108 - 17125603
3	B-IGH	B-IGHV3-80 [P]	--	--	--	--	17132447 - 17132955

[a] Contig NC_072496.1; [b] Functional rearranged in ≥ 1 individual in bulk analysis; [c] Functional rearranged in ≥ 1 individual in single cell analysis; [P] Pseudogene; [ORF] Open reading frame; [d] Putative allele found in ≥ 1 individual

Table S6 (*continued*)

Family	Locus	Proposed designation	Expressed	Allele	Human homolog	Homolog identity	Genome Position[a]
3	B-IGH	B-IGHV3-81 P	--	--	--	--	17145105 - 17145439
3	B-IGH	B-IGHV3-82 P	--	--	--	--	17147532 - 17147986
3	B-IGH	B-IGHV3-83 [P]	--	--	--	--	17154787 - 17155121
3	B-IGH	B-IGHV3-86 [ORF]	Y[b,c]	--	IGHV3-30-5	91.5	17175826 - 17176321
3	B-IGH	B-IGHV3-90	Y[b,c]	--	IGHV3-48	86.8	17215824 - 17216311
3	B-IGH	B-IGHV3-91	Y[b,c]	--	IGHV3-23	85.1	17243422 - 17243916
3	B-IGH	B-IGHV3-93 [P]	--	--	--	--	17252274 - 17252571
3	B-IGH	B-IGHV3-94	Y[b,c]	--	IGHV3-33	91.4	17258745 - 17259242
3	B-IGH	B-IGHV3-95	Y[b,c]	Y	IGHV3-71	84.8	17280061 - 17280555
4	B-IGH	B-IGHV4-3	Y[b,c]	Y	IGHV4-4	81.4	16515583 - 16515972
4	B-IGH	B-IGHV4-5 [P]	--	--	--	--	16536112 - 16538064
4	B-IGH	B-IGHV4-6	Y[b,c]	Y	IGHV4-28	86.8	16541640 - 16542028
4	B-IGH	B-IGHV4-11	Y[b,c]	--	IGHV4-39	85.0	16579921 - 16580313
4	B-IGH	B-IGHV4-13	Y[b]	--	IGHV4-4	84.1	16586700 - 16587088
4	B-IGH	B-IGHV4-15	Y[b,c]	--	IGHV4-38-2	82.4	16615433 - 16615822
4	B-IGH	B-IGHV4-16 [P]	--	--	--	--	16622277 - 16622278
4	B-IGH	B-IGHV4-23 [P]	--	--	--	--	16656563 - 16656998
4	B-IGH	B-IGHV4-31 [P]	--	--	--	--	16725032 - 16725033
4	B-IGH	B-IGHV4-32	Y[b]	--	IGHV4-28	81.9	16731799 - 16732191
4	B-IGH	B-IGHV4-34 [P]	--	--	--	--	16736829 - 16737222
4	B-IGH	B-IGHV4-35 [P]	--	--	--	--	16747513 - 16747607
4	B-IGH	B-IGHV4-38	N	--	IGHV4-28	80.7	16772220 - 16772606
4	B-IGH	B-IGHV4-39 [P]	--	--	--	--	16796802 - 16796918
4	B-IGH	B-IGHV4-40	Y[b,c]	Y	IGHV4-39	78.6	16807104 - 16807498
4	B-IGH	B-IGHV4-47 [P]	--	--	--	--	16874859 - 16875153
4	B-IGH	B-IGHV4-52 [P]	--	--	--	--	16908253 - 16908635
4	B-IGH	B-IGHV4-57 [P]	--	--	--	--	16943608 - 16943970
4	B-IGH	B-IGHV4-60 [P]	--	--	--	--	16959989 - 16960423
4	B-IGH	B-IGHV4-63	Y[b,c]	--	IGHV4-30-2	81.1	16975868 - 16976344
4	B-IGH	B-IGHV4-65	Y[b,c]	--	IGHV4-38-2	82.1	16994204 - 16994681
4	B-IGH	B-IGHV4-71[P]	--	--	--	--	17036667 - 17037020
4	B-IGH	B-IGHV4-73	Y[b,c]	--	IGHV4-30-2	81.8	17053219 - 17053694
4	B-IGH	B-IGHV4-78[P]	--	--	--	--	17120700 - 17121032
4	B-IGH	B-IGHV4-85 [P]	--	--	--	--	17167544 - 17167884
4	B-IGH	B-IGHV4-87 [P]	--	--	--	--	17180661 - 17181023
4	B-IGH	B-IGHV4-89 [P]	--	--	--	--	17194181 - 17194492
4	B-IGH	B-IGHV4-92	Y[b]	--	IGHV4-39	82.3	17245898 - 17246375
4	B-IGH	B-IGHV4-97 [P]	--	--	--	--	17292871 - 17293407
4	B-IGH	B-IGHV4-98 [P]	--	--	--	--	17296958 - 17297686
5	B-IGH	B-IGHV5-44 [P]	--	--	--	--	16835449 - 16835450
5	B-IGH	B-IGHV5-53 [P]	--	--	--	--	16919228 - 16918916
6	B-IGH	B-IGHV6-2	Y[b,c]	--	IGHV6-1	81.5	16505787 - 16506187
6	B-IGH	B-IGHV6-9	N	--	IGHV6-1	83.6	16564103 - 16564503
6	B-IGH	B-IGHV6-18	Y[b,c]	Y	IGHV6-1	84.9	16627033 - 16627433
6	B-IGH	B-IGHV6-21 [P]	--	--	--	--	16641247 - 16641342
6	B-IGH	B-IGHV6-25 [P]	--	--	--	--	16670778 - 16671178
7	B-IGH	B-IGHV7-45	Y[b,c]	--	IGHV7-4-1	81.9	16840998 - 16841387
7	B-IGH	B-IGHV7-54	Y[b,c]	Y	IGHV7-4-1	84.7	16924172 - 16924561
7	B-IGH	B-IGHV7-66	Y[b,c]	--	IGHV7-4-1	80.7	17014838 - 17015314

[a] Contig NC_072496.1; [b] Functional rearranged in ≥ 1 individual in bulk analysis; [c] Functional rearranged in ≥ 1 individual in single cell analysis; [P] Pseudogene; [ORF] Open reading frame; [d] Putative allele found in ≥ 1 individual

Table S7. IGLV gene annotations

Family	Proposed designation	Orientation	Expressed	Allele	Genome Position[a]
1	IGLV1-1	R	Y[b,c]	--	542686 - 524186
1	IGLV1-2	R	Y[b,c]	--	527862 - 527362
1	IGLV1-3	R	Y[b,c]	--	521727 - 521230
1	IGLV1-4	R	Y[b,c]	--	517896 - 517396
1	IGLV1-5	R	Y[b,c]	--	514165 - 513665
1	IGLV1-6	R	Y[b,c]	--	510669 - 510169
1	IGLV1-7	R	Y[b,c]	--	506960 - 506460
1	IGLV1-8	R	Y[b,c]	--	503693 - 503192
1	IGLV1-9	R	Y[b,c]	--	500099 - 499599
1	IGLV1-58	F	Y[b,c]	--	228608 - 229122
1	IGLV1-85 [P]	F	--	--	146790 - 147127
1	IGLV1-90	F	Y[b,c]	--	131365 - 131865
1	IGLV1-96 [P]	F	--	--	113044 - 113543
1	IGLV1-102 [P]	F	Y[b,c]	--	94768 - 95272
1	IGLV1-109	F	Y[b,c]	Y[d]	75840 - 76332
1	IGLV1-113 [ORF]	F	N	--	62179 - 62760
1	IGLV1-118	F	Y[b,c]	--	46509 - 47009
1	IGLV1-123	F	Y[b,c]	--	31165 - 31668
2	IGLV2-34	F	Y[b,c]	--	334709 - 335212
2	IGLV2-35	F	Y[b,c]	--	332119 - 332623
2	IGLV2-36	F	Y[b,c]	--	329309 - 329812
2	IGLV2-37	F	Y[b,c]	--	326835 - 327329
2	IGLV2-38	F	Y[b,c]	--	324333 - 324835
2	IGLV2-39	F	Y[b,c]	--	321654 - 322157
2	IGLV2-40	F	Y[b,c]	--	319132 - 319635
2	IGLV2-41	F	Y[b,c]	--	316529 - 317033
2	IGLV2-42 [P]	F	--	--	313888 - 314392
2	IGLV2-43	F	Y[b,c]	--	308764 - 309267
2	IGLV2-44	F	Y[b,c]	--	302766 - 303268
2	IGLV2-45	F	Y[b,c]	--	300751 - 301263
2	IGLV2-46	F	Y[b,c]	Y[d]	296116 - 296626
2	IGLV2-47	F	Y[b,c]	--	292268 - 292771
2	IGLV2-48	F	Y[b,c]	--	288407 - 288910
3	IGLV3-10	F	Y[b,c]	--	417299 - 418041
3	IGLV3-11	F	Y[b]	--	413465 - 414191
3	IGLV3-12	F	Y[b,c]	--	409398 - 409902
3	IGLV3-14	F	Y[b,c]	--	402436 - 402994
3	IGLV3-16	F	Y[b,c]	Y	396020 - 396599
3	IGLV3-18	F	Y[b,c]	--	389289 - 390007
3	IGLV3-19	F	Y[b,c]	--	385460 - 385963
3	IGLV3-21	F	Y[b,c]	--	378575 - 379294
3	IGLV3-22 [ORF]	F	Y[b,c]	--	375576 - 376071
3	IGLV3-24 [P]	F	--	--	369261 - 369983
3	IGLV3-26	F	Y[b,c]	--	362481 - 363201
3	IGLV3-28	F	Y[b,c]	--	355877 - 356459
3	IGLV3-30	F	Y[b,c]	--	349573 - 350017
3	IGLV3-31	F	Y[b,c]	--	345418 - 345923
3	IGLV3-33 [ORF]	F	Y[b,c]	--	338573 - 339217
4	IGLV4-13	F	Y[b,c]	--	405385 - 405907
4	IGLV4-15	F	Y[b,c]	--	398776 - 399300
4	IGLV4-17	F	Y[b,c]	--	392204 - 392710

[a] Contig NC_072495.1; [b] Functional rearranged in ≥ 1individual in bulk analysis; [c] Functional rearranged in ≥ 1 individual in single cell analysis; [P] Pseudogene; [ORF] Open reading frame; [d] Putative allele found in ≥ 1 individual

Table S7. (*continued*)

Family	Proposed designation	Orientation	Expressed	Allele	Genome Position[a]
4	IGLV4-20	F	Y[b,c]	--	381497 - 382020
4	IGLV4-23	F	Y[b,c]	--	372164 - 372687
4	IGLV4-25	F	Y[b,c]	--	365379 - 365892
4	IGLV4-27	F	Y[b,c]	--	358582 - 359095
4	IGLV4-29 [P]	F	--	--	352208 - 352732
4	IGLV4-32	F	Y[b,c]	--	341429 - 341954
5	IGLV5-59	F	Y[b,c]	--	224965 - 225477
5	IGLV5-70	F	Y[b,c]	--	188555 - 189068
5	IGLV5-71	F	Y[b,c]	--	185817 - 186319
5	IGLV5-75	F	Y[b,c]	--	173882 - 174405
5	IGLV5-76	F	Y[b,c]	--	170811 - 171324
5	IGLV5-77	F	Y[b,c]	--	167959 - 168480
5	IGLV5-79	F	Y[b,c]	--	163593 - 164106
5	IGLV5-80	F	Y[b,c]	--	160736 - 161244
5	IGLV5-83	F	Y[b,c]	--	152368 - 152889
5	IGLV5-84	F	Y[b,c]	--	150314 - 150819
5	IGLV5-86	F	Y[b,c]	--	144917 - 145425
5	IGHV5-88	F	Y[b,c]	--	137345 - 137866
5	IGLV5-89	F	Y[b,c]	--	134939 - 135452
5	IGLV5-92	F	Y[b,c]	--	126604 - 127125
5	IGLV5-93	F	Y[b,c]	--	123527 - 124040
5	IGLV5-94	F	Y[b,c]	--	119640 - 120156
5	IGLV5-95	F	Y[b,c]	--	115797 - 116310
5	IGLV5-97	F	Y[b,c]	Y	109831 - 110336
5	IGLV5-98	F	Y[b,c]	--	107266 - 107787
5	IGLV5-99	F	Y[b]	--	104857 - 105370
5	IGLV5-101 [P]	F	--	--	97522 - 98034
5	IGLV5-104	F	Y[b,c]	--	89861 - 90369
5	IGLV5-105	F	Y[b,c]	--	86270 - 86783
5	IGLV5-107	F	Y[b,c]	--	80726 - 81239
5	IGLV5-108	F	Y[b,c]	--	78643 - 79167
5	IGLV5-112	F	Y[b,c]	--	65965 - 66467
5	IGLV5-114	F	Y[b,c]	--	60704 - 61206
5	IGLV5-115	F	Y[b,c]	--	55974 - 56498
5	IGLV5-116	F	Y[b,c]	--	53074 - 53587
5	IGLV5-117	F	Y[b,c]	--	50719 - 51249
5	IGLV5-120	F	Y[b,c]	--	40205 - 40707
5	IGLV5-121	F	Y[b,c]	--	37102 - 37617
5	IGLV5-122	F	Y[b,c]	--	33917 - 34430
5	IGLV5-125	F	Y[b]	--	26055 - 26563
5	IGLV5-126	F	Y[b,c]	--	22962 - 23483
5	IGLV5-127	F	Y[b,c]	--	20863 - 21401
6	IGLV6-82	F	Y[b,c]	--	155978 - 156497
6	IGLV6-87	F	Y[b,c]	--	140979 - 141490
7	IGLV7-49 [ORF]	F	Y[b,c]	--	259071 - 259571
7	IGLV7-50 [ORF]	F	Y[b,c]	--	256936 - 257427
7	IGLV7-51	F	Y[b,c]	--	253269 - 253741
7	IGLV7-103	F	Y[b,c]	--	91817 - 92289
7	IGLV7-110	F	Y[b,c]	--	72422 - 72891
7	IGLV7-111	F	Y[b,c]	--	69163 - 69635
7	IGLV7-119	F	Y[b]	--	43601 - 43944
7	IGLV7-124	F	Y[b,c]	--	28058 - 28486

[a] Contig NC_072495.1; [b] Functional rearranged in ≥ 1 individual in bulk analysis; [c] Functional rearranged in ≥ 1 individual in single cell analysis; [P] Pseudogene; [ORF] Open reading frame; [d] Putative allele found in ≥ 1 individual

Table S7. (*continued*)

Family	Proposed designation	Orientation	Expressed	Allele	Genome Position[a]
8	IGLV8-52	F	Y[b,c]	--	249450 - 249945
8	IGLV8-53	F	Y[b,c]	--	244201 - 244692
8	IGLV8-55	F	Y[b,c]	Y	238081 - 238572
8	IGLV8-57	F	Y[b,c]	--	231949 - 232440
8	IGLV8-63	F	Y[b,c]	--	212659 - 213150
8	IGLV8-64	F	Y[b,c]	--	208163 - 208654
8	IGLV8-66	F	Y[b,c]	--	202018 - 202509
8	IGLV8-68	F	Y[b,c]	--	195541 - 196032
8	IGLV8-73	F	Y[b,c]	--	179972 - 180464
8	IGLV8-74	F	Y[b,c]	--	177391 - 177886
10	IGLV10-54	F	Y[b,c]	--	240901 - 241415
10	IGLV10-56	F	Y[b,c]	--	234752 - 235266
10	IGLV10-65	F	Y[b,c]	--	204841 - 205355
10	IGLV10-67	F	Y[b,c]	--	198349 - 198863
10	IGLV10-69	F	Y[b,c]	--	192184 - 192698
11	IGLV11-60	F	Y[b,c]	--	222174 - 222676
11	IGLV11-61	F	Y[b,c]	Y	218731 - 219213
11	IGLV11-72	F	Y[b,c]	--	182415 - 182892
11	IGLV11-78	F	Y[b,c]	--	166001 - 166522
11	IGLV11-81	F	Y[b,c]	--	158775 - 159296
11	IGLV11-91	F	Y[b,c]	--	128771 - 129248
11	IGLV11-100	F	Y[b,c]	--	101323 - 101838
11	IGLV11-106	F	Y[b,c]	--	84310 - 84831

[a] Contig NC_072495.1; [b] Functional rearranged in ≥ 1 individual in bulk analysis; [c] Functional rearranged in ≥ 1 individual in single cell analysis; [P] Pseudogene; [ORF] Open reading frame; [d] Putative allele found in ≥ 1 individual

Table S8. IGLJ gene annotations

Designation	Genome Position [a]
IGLJ1	428677 - 428714
IGLJ2	433077 - 433114
IGLJ3	436484 - 436521
IGLJ4	439880 - 439917
IGLJ5	443353 - 443390
IGLJ6	446811 - 446848
IGLJ7	450345 - 450382
IGLJ8	453828 - 453865
IGLJ9	457339 - 457376
IGLJ10	460870 - 460907
IGLJ11	464355 - 464392
IGLJ12	467846 - 467883

[a] Contig NC_072495.1

Table S9. IGLC gene annotations

Designation	Genome Position [a]
IGLC1	430162 - 430480
IGLC2	434420 - 434738
IGLC3	437795 - 438113
IGLC4	441223 - 441541
IGLC5	444668 - 444987
IGLC6	448188 - 448506
IGLC7	451667 - 451985
IGLC8	455178 - 455496
IGLC9	458710 - 459028
IGLC10	462192 - 462510
IGLC11	465680 - 465998
IGLC12	469099 - 469417

[a] Contig NC_072495

Table S10: Genbank accession numbers for the genomes and IGHM loci used to reconstruct the history of the IGH duplication, depicted in Figure 2

Species	Genome Accession	Locus	Sequence Accession	Chromosome
Miniopteridae				
Miniopterus fuliginosus	GCA_051201465.1		JBLYEM010000026	
Miniopterus natalensis	GCF_001595765.1		NW15504874.1	
Miniopterus schreibersii	GCA_964146895.2		OZ071092.2	Chr 1
Molossidae				
Tadarida brasiliensis	GCA_030848825.1		CM061257	Chr 1
Phyllostomidae				
Artibeus jamaicensis			PTG000026L	
Phyllostomus discolor	GCA_004126475.3		NC_040917.2	Chr 15
Pteropodidae				
Eonycteris spelaea	GCA_003508835.1		PUFA01000875	
Rhinolophidae				
Rhinolophus ferrumequinum	GCF_004115265.2		NC46289	Chr 6
Vespertilionidae				
Antrozous pallidus	GCA_027563665.1	A-IGH*	CM050515	Chr 8
Cnephaeus nilssonii	GCA_030846915.1	A-IGH	JAULJE010000009	
		B-IGH	JAULJE010000024	
Corynorhinus townsendii	GCA_026230045.1	A-IGH	JAPDVU010000001	
		B-IGH	JAPDVU010000016	
Eptesicus fuscus	GCA_027574615.1	A-IGH	CM050732	Chr 5
		B-IGH	CM050751	Chr 24
Ia io	GCA_025583905.1	A-IGH	JAJQQW010000008	Chr 7
		B-IGH	JAJQQW010000024	Chr 23
Lasiurus cinereus	GCA_011751065.5	A-IGH	JAAGEH040000943.1	
		B-IGH	JAAGEH040000014.1	
Murina feae	GCA_004026665	A-IGH	PVJC01049261.1	
		B-IGH	PVJC01060259.1	
Myotis brandtii	GCF_000412655	A-IGH	NW5353568	
		B-IGH	NW5370397.1	
Myotis daubentonii	GCA_963242275.2	A-IGH	OZ250116.1	Chr 1
		B-IGH	OZ250136.1	Chr 20
Myotis emarginatus	GCA_965115925.2	A-IGH	OZ220647.2	Chr 1
		B-IGH	OZ220668.2	Chr 21
Myotis findleyi	GCA_038630795.1	A-IGH	JAWQEE010059017	
		B-IGH	JAWQEE010000008	
Myotis lucifugus carissima	GCA_048340685.1	A-IGH	CM107823	Chr V1
		B-IGH	CM107843	Chr V25
Myotis myotis	GCA_004026985.1	A-IGH	PVIZ010033222	
		B-IGH	PVIZ010106845	
Myotis mystacinus	GCA_964195625.1	A-IGH	OZ077851	Chr 1
		B-IGH	OZ077871	Chr 21
Myotis nattereri	GCA_964212035.2	A-IGH	OZ125675.2	Chr 1
		B-IGH	OZ125696.2	Chr 20
Myotis pilosus	GCA_036010255.1	A-IGH	JASKON010000004.1	
		B-IGH	JASKON010000027.1	

Table S10. (*continued*)

Species	Genome Accession	Locus	Sequence Accession	Chromosome
Myotis vivesi	GCA_035771395.1	A-IGH	JAWPEG010064502	
		B-IGH	JAWPEG010038709	
Myotis yumanensis	GCA_028538775.1	A-IGH	JAPQVT010000001	
		B-IGH	JAPQVT010000022	
Nyctalus aviator	GCA_036971965.2	A-IGH	CM072922	Chr 1
		B-IGH	CM072940	Chr 19
Nyctalus leisleri	GCA_964264875.2	A-IGH	OX183624.2	Chr 1
		B-IGH	CAXWAE020000002.1	
Pipistrellus abramus	GCA_044885105.1	A-IGH	CM097868.1	Chr 1
		B-IGH	CM097869.1	Chr 2
Pipistrellus hanaki	GCA_964339955.5	A-IGH	OZ03233.1	Chr 1
		B-IGH	OZ03252.1	Chr 20
Pipistrellus kuhlii	GCF_014108245.1	A-IGH	NW23425413	
		B-IGH	NW23425588	
Pipistrellus nathusii	GCA_963693515.1	A-IGH	OY882858	Chr 1
		B-IGH	OY882875	Chr 18
Pipistrellus pipistrellus	GCA_903992545.1	A-IGH	LR862356	Chr 1
		B-IGH	LR862376	Chr 20
Pipistrellus pygmaeus	GCA_949987765.2	A-IGH	CATLKD010000043	
		B-IGH	CATLKD010000165	
Plecotus auritus	GCA_963455305.1	A-IGH	OY734022	Chr 1
		B-IGH	OY734036	Chr 14

* *Antrozous pallidus* has a single IGH locus, but it clusters phylogenetically with the A-IGH locus of other vespertilionids.

Table S11. Summary of single cell numbers for used for analysis

Bat ID	Cell Type	Isotype	Locus	Absolute cell number
217	Antibody secreting cells	IGHA	A-IGH	24
217	Antibody secreting cells	IGHG	A-IGH	39
217	Antibody secreting cells	IGHM	A-IGH	5
217	Germinal center cells	IGHG	A-IGH	4
217	Memory B cells	IGHA	A-IGH	26
217	Memory B cells	IGHG	A-IGH	32
217	Memory B cells	IGHD	A-IGH	1
217	Memory B cells	IGHM	A-IGH	35
217	Naïve B cells	IGHM	A-IGH	27
217	Antibody secreting cells	IGHG	B-IGH	17
217	Antibody secreting cells	IGHM	B-IGH	6
217	Memory B cells	IGHA	B-IGH	2
217	Memory B cells	IGHG	B-IGH	8
217	Memory B cells	IGHM	B-IGH	17
217	Naïve B cells	IGHM	B-IGH	22
231	Antibody secreting cells	IGHA	A-IGH	10
231	Antibody secreting cells	IGHG	A-IGH	284
231	Antibody secreting cells	IGHM	A-IGH	41
231	Germinal center cells	IGHG	A-IGH	6
231	Germinal center cells	IGHM	A-IGH	3
231	Memory B cells	IGHA	A-IGH	16
231	Memory B cells	IGHE	A-IGH	1
231	Memory B cells	IGHG	A-IGH	196
231	Memory B cells	IGHD	A-IGH	7
231	Memory B cells	IGHM	A-IGH	151
231	Naïve B cells	IGHD	A-IGH	22
231	Naïve B cells	IGHM	A-IGH	833
231	Antibody secreting cells	IGHA	B-IGH	3
231	Antibody secreting cells	IGHG	B-IGH	110
231	Antibody secreting cells	IGHM	B-IGH	45
231	Germinal center cells	IGHG	B-IGH	2
231	Memory B cells	IGHA	B-IGH	5
231	Memory B cells	IGHG	B-IGH	115
231	Memory B cells	IGHM	B-IGH	95
231	Naïve B cells	IGHM	B-IGH	169
567	Antibody secreting cells	IGHA	A-IGH	6
567	Antibody secreting cells	IGHG	A-IGH	53
567	Antibody secreting cells	IGHM	A-IGH	38
567	Germinal center cells	IGHA	A-IGH	1
567	Germinal center cells	IGHG	A-IGH	12
567	Germinal center cells	IGHM	A-IGH	2
567	Memory B cells	IGHA	A-IGH	14
567	Memory B cells	IGHG	A-IGH	79
567	Memory B cells	IGHD	A-IGH	6
567	Memory B cells	IGHM	A-IGH	461
567	Naïve B cells	IGHD	A-IGH	6
567	Naïve B cells	IGHM	A-IGH	363
567	Antibody secreting cells	IGHA	B-IGH	2
567	Antibody secreting cells	IGHG	B-IGH	20
567	Antibody secreting cells	IGHM	B-IGH	14
567	Germinal center cells	IGHG	B-IGH	3
567	Germinal center cells	IGHM	B-IGH	1
567	Memory B cells	IGHA	B-IGH	4

567	Memory B cells	IGHG	B-IGH	15

Table S11. *continued*

Bat ID	Cell Type	Isotype	Locus	Absolute cell number
567	Memory B cells	IGHM	B-IGH	147
567	Naïve B cells	IGHM	B-IGH	129
2007	Antibody secreting cells	IGHA	A-IGH	98
2007	Antibody secreting cells	IGHG	A-IGH	496
2007	Antibody secreting cells	IGHM	A-IGH	18
2007	Germinal center cells	IGHG	A-IGH	4
2007	Memory B cells	IGHA	A-IGH	22
2007	Memory B cells	IGHE	A-IGH	2
2007	Memory B cells	IGHG	A-IGH	97
2007	Memory B cells	IGHD	A-IGH	1
2007	Memory B cells	IGHM	A-IGH	21
2007	Naïve B cells	IGHD	A-IGH	2
2007	Naïve B cells	IGHM	A-IGH	56
2007	Antibody secreting cells	IGHA	B-IGH	8
2007	Antibody secreting cells	IGHE	B-IGH	1
2007	Antibody secreting cells	IGHG	B-IGH	305
2007	Antibody secreting cells	IGHM	B-IGH	28
2007	Germinal center cells	IGHG	B-IGH	1
2007	Memory B cells	IGHA	B-IGH	7
2007	Memory B cells	IGHG	B-IGH	54
2007	Memory B cells	IGHM	B-IGH	22
2007	Naïve B cells	IGHM	B-IGH	12

Table S12. Primers sequences for immunoglobulin repertoire amplification

Primer Name	Sequence
Epfu-IgM-Bulk-R1	GATTACGCCAAGCTTCTTAAAGCTCCAGGAGAAGGTGACGGAG
Epfu-IgG-Bulk-R1	TCCGGGAAGTAGCCAGAGACCA
Epfu-IgL-Bulk-R1	GGGTGGCCTTGTTCTTGCTGAGCTCCT
Epfu-IgM-Bulk-R2	TCACAGGAGACGAGGGGGAAGA
Epfu-IgG-Bulk-R2	CAGAGAYCAGGCAGCCCAGGGASAYC
Epfu-IgL-Bulk-R2	GCGGAKGGCGGGAACAGAGACACY
Epfu-IgM-10x-R1	TCGCCGTTGGAGTGTTTGACCT
Epfu-IgG-10x-R1	GGCACCGTCACCATGCTGCT
Epfu-IgA-10x-R1	GGCACGTCCTTGGTCTGGCT
Epfu-IgD-10x-R1	CTGGCCACTGGAAGGTCTTGGAC
Epfu-IgE-10x-R1	GGTGAACCGCTGTTTGGCCC
Epfu-IgL-10x-R1	GGTCTTCCCGTCGTGCGTGA
Epfu-IgM-10x-R2	GGAGTCGGGCAGGAAGTCCTTG
Epfu-IgG-10x-R2	TCCGGGAAGTAGCCAGAGACCA
Epfu-IgA-10x-R2	CTGGACCAGGCAGCCGATGA
Epfu-IgD-10x-R2	WCACGCCTGCTTTTGGGGCCTT
Epfu-IgE-10x-R2	TTTGGAGCAGGGGGCCAAGG
Epfu-IgL-10x-R2	GGGTGGCCTTGTTCTTGCTGAG

Supplementary Data File Captions:

File S1. Genetic alignment of IGHM sequences from publicly available bat genomes. Alignment of genetic regions containing IGHM exons and introns for phylogenetic inference.

File S2. Consensus PhyML phylogenetic hypothesis of IGHM sequences. Final consensus phylogenetic hypothesis for the relationships between IGHM sequences (File S1) including bootstrap values (out of 100).

File S3. IGH Allele Evaluation. (A) IGHV variant calls at the A-IGH and B-IGH loci in six individuals, including mutation strings, clone counts, somatic hypermutation rates, expressed isotypes, and bulk/single-cell concordance; **(B)** single-cell detection status and isotype breadth for all reference IGHV genes, **(C)** per-bat allele counts by gene, and **(D)** a gene-by-bat crosstab of novel allele counts in bulk and single-cell data, with "R" indicating reference gene-only and "—" indicating no expression.
